# Small RNAs and the competing endogenous RNA network in high grade serous ovarian cancer tumor spread

**DOI:** 10.18632/oncotarget.9243

**Published:** 2016-05-09

**Authors:** Anna Bachmayr-Heyda, Katharina Auer, Nyamdelger Sukhbaatar, Stefanie Aust, Simon Deycmar, Agnes T. Reiner, Stephan Polterauer, Sabine Dekan, Dietmar Pils

**Affiliations:** ^1^ Department of Obstetrics and Gynecology, Comprehensive Center Center (CCC), Medical University of Vienna, Vienna, Austria; ^2^ Department of Pathology, Medical University of Vienna, Vienna, Austria

**Keywords:** ovarian cancer, peritoneal tumor spread, small RNA sequencing, competing endogenous RNA network, prognostic signature

## Abstract

High grade serous ovarian cancer (HGSOC) is among the most deadly malignancies in women, frequently involving peritoneal tumor spread. Understanding molecular mechanisms of peritoneal metastasis is essential to develop urgently needed targeted therapies. We described two peritoneal tumor spread types in HGSOC apparent during surgery: miliary (numerous millet-sized implants) and non-miliary (few big, bulky implants). The former one is defined by a more epithelial-like tumor cell characteristic with less immune cell reactivity and with significant worse prognosis, even if corrected for typical clinicopathologic factors.

23 HGSOC patients were enrolled in this study. Isolated tumor cells from fresh tumor tissues of ovarian and peritoneal origin and from ascites were used for ribosomal RNA depleted RNA and small RNA sequencing. RT-qPCR was used to validate results and an independent cohort of 32 patients to validate the impact on survival. Large and small RNA sequencing data were integrated and a new gene-miRNA set analysis method was developed.

Thousands of new small RNAs (miRNAs and piwi-interacting RNAs) were predicted and a 13 small RNA signature was developed to predict spread type from formalin-fixed paraffin-embedded tissues. Furthermore, integrative analyses of RNA sequencing and small RNA sequencing data revealed a global upregulation of the competing endogenous RNA network in tumor tissues of non-miliary compared to miliary spread, *i.e*. higher expression of circular RNAs and long non-coding RNAs compared to coding RNAs but unchanged abundance of small RNAs. This global deregulated expression pattern could be co-responsible for the spread characteristic, miliary or non-miliary, in ovarian cancer.

## INTRODUCTION

Epithelial ovarian cancer (EOC) is the second most common and the most deadly gynecological cancer with a 5a-survival rate of only 30% in advanced stages [1, 2]. In this study only high grade serous ovarian cancer (HGSOC) patients (60–70% of EOC) were included. Despite the heterogeneity of HGSOC, patients are usually treated with standard therapy (cytoreductive surgery and carboplatin-based chemotherapy). HGSOC is often complicated by peritoneal involvement and accumulation of malignant peritoneal fluid, *i.e.* ascites. Unlike in other cancer entities, most patients suffering from HGSOC die from consequences of peritoneal tumor spread, whereas distant metastases are less important. Better understanding of the mechanisms underlying HGSOC and especially the mechanisms for peritoneal tumor spread are urgently needed.

MicroRNAs (miRNAs) are non-coding RNAs (ncRNAs) (18–23 nucleotides (nt) long) and (mostly down-) regulate gene expression by sequence-specific binding of their target mRNAs. They are involved in several pathologies including ovarian cancer [3, 4]. The term competing endogenous RNA (ceRNA) network describes the several different RNA species which compete for the binding of miRNAs including mRNAs, long non-coding RNAs (lncRNAs), and circular RNAs (circRNAs). The role of the ceRNA network in cancer progression has previously been reviewed [5]. miRNAs are also discussed as prognostic and diagnostic biomarkers or drug targets in cancer therapy [6, 7]. Piwi-interacting RNAs (piRNAs) are also regulatory ncRNAs (26–32 nt) [8]. One of their major functions seems to be in germline development. However, evidence for a role of piRNAs also in cancer has been suggested [9, 10].

We recently published a study on RNA-sequencing (RNA-seq) and flow cytometry data of enriched HGSOC tumor cells [11] to which we now present the matched small RNA-seq (sRNA-seq, < 200 nt) data. We introduced a novel classification criterion for HGSOC patients concerning the pattern of peritoneal tumor spread, *i.e.* miliary (widespread, millet-like lesions with a worse overall survival (OS)) versus non-miliary (few exophytically growing, bigger implants with a better prognosis). In the current study, we assess global expression differences including the small transcriptome between HGSOC patients characterized by these two different modes of peritoneal tumor spread.

## RESULTS

### Patients, samples, and experimental design

We are the first to study the complete transcriptome of enriched HGSOC cells from spatially different tissue origins from 23 patients (solid tumors: (P) primary/ovarian and (M) metastatic/peritoneal and from ascites: (A) ascitic single cells and (S) spheroids, defined as cell aggregates between 30 and 150 μm, see Supplementary methods). 22 of them (95.7%) carried a functional tumor protein 53 (TP53) mutation. Most of the patients presented with International Federation of Gynecology and Obstetrics (FIGO) III, two with FIGO II, and one with FIGO IV. The median age at diagnosis was 54 years (34–81, Supplementary Table S1). Eleven patients presented with miliary peritoneal tumor spread; twelve patients with non-miliary peritoneal tumor spread (four without any peritoneal involvement at all (two lymph node positive) and eight with few big, bulky peritoneal implants). Patients whose peritoneal tumor spread type could not be determined were excluded. Supplementary Figure S1 outlines the used tissue samples and the two different spread types.

Two major objectives were pursued in this work: i) The first was to understand the role of small ncRNAs (miRNAs and piRNAs) and of the ceRNA network in HGSOC tumor development, especially regarding differences between the two different modes of peritoneal tumor spread, miliary and non-miliary. ii) The second aim was to develop and validate a small RNA signature applicable to formalin-fixed paraffin-embedded (FFPE) tissues to diagnose these tumor spread types. Major assets of our approach are the matched long rRNA-depleted RNA and complete small RNA sequencing data, thus interrogating the complete transcriptome, of microenvironment-free (*i.e.* positively selected) cancer cells from spatial separated tumor tissues and the integrative analyses of major players of the ceRNA network. For purpose i) the small RNAome was defined, differentially expressed single small RNAs (sRNAs) were analyzed, a new combined gene-miR set analysis method was developed, gene targets of selected differentially expressed miRNAs were biologically interpreted, and finally differences between both spread types in global abundances of main RNA species of the ceRNA network were assessed. For purpose ii) we developed or used pre-developed RT-qPCR systems for selected candidate sRNAs (miRNAs and piRNAs) and normalizers to validate the expression using RNA isolated from fresh tissues and matched FFPE tissues of the training cohort and also from FFPE tissues of an independent validation cohort. A robust sRNA signature was developed and successfully validated, showing again a negative and independent impact of the miliary spread type on overall survival.

### Small RNA-sequencing

**Figure 1:**
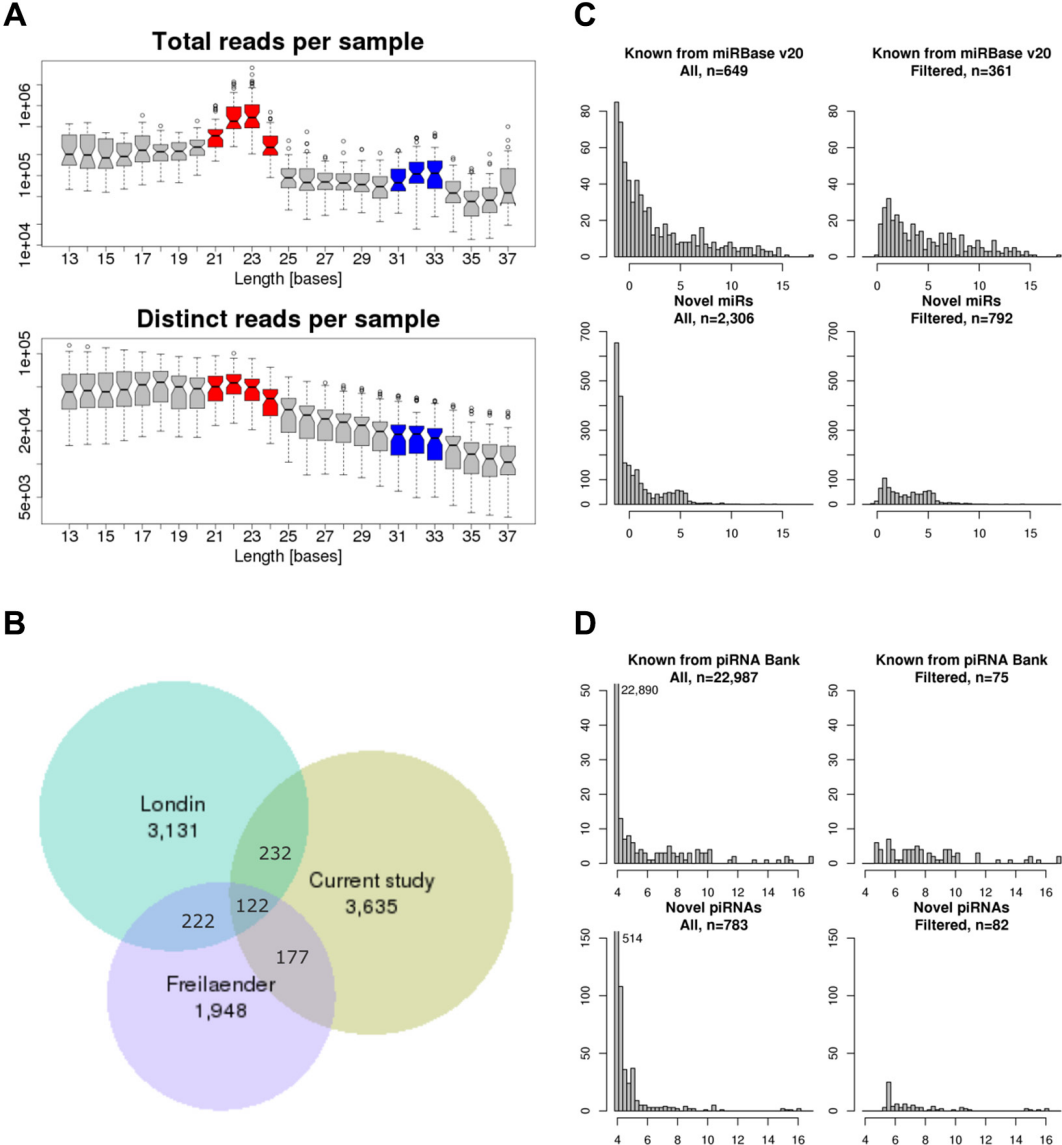
Small RNA-sequencing (**A**) Boxplots of read length distribution of total (upper) and distinct (lower) reads per sample, red: miRNA peak 21–24 nucleotides, blue: piRNA peak 31–33 nucleotides. (**B**) Venn diagram comparing novel predicted miRNAs of the current study with two other recently published studies on miRNAs [13, 14]. (**C**) Frequency distributions of the median number of supporting reads for all known and predicted miRNAs (left) and for only those with > 100 supporting reads among all samples (right). (**D**) Frequency distributions of the median number of supporting reads for all known and predicted piRNAs (left) and for only those with > 100 supporting reads among all samples (right). (C and D) x-axis: cyclic loess normalized log_2_((gene read count + 0.5)/millions of total library read counts).

In total, 43 samples passed quality control: 11 P, 11 M, 11 A, and 10 S. sRNAseq yielded a median read depth of 14.2 (10.7–25.1) million reads. The length distribution of trimmed reads revealed a prominent peak at 21–24 nt (putative miRNAs) and a smaller peak at 31–33 nt (putative piRNAs,). As expected, the length distribution of unique sequences showed no clear peaks (Figure 1A).

Reads were annotated to 768 previously known miRNA loci (miRBase v20 [12]) and using a computational miRNA prediction approach, *i.e.* miRDeep*, 4,166 novel miRNA loci (stem loops) were predicted (thereof 241 new loci for 38 already known miRNAs; for chromosomal visualization see Supplementary Figure S8). Newly predicted miRNAs were named “novel miR-“. The comparison of predicted miRNAs to those of two other recent studies revealed only a small overlap of 3.4% among all three studies [13, 14] (Figure 1B).

piRNAs were predicted from all reads with proTRAC (*cf*. Supplementary Figure S2 showing all piRNA clusters) and intersected with known piRNAs and accordingly annotated. Predicted piRNAs were named “novel piR-“, followed by the number of their piRNA cluster, the chromosome, and the starting position (*e.g.* piR-n9_chr19_12814412). For statistical analyses miRNA and piRNA loci yielding the same or similar mature mi/piRNAs were collapsed and only those with > 100 supporting reads among all samples were included (361 known and 792 novel miRNAs, Figure 1C and 1D, Supplementary Table S2).

### Differential sRNA expression analyses

Differential miRNA and piRNA expression analyses between the ascitic samples, A and S, and between the solid tumor samples, P and M, revealed very low numbers of differentially expressed sRNAs. Thus, ascitic A/S- and solid P/M-tissue samples were analyzed together but information of tissue origin was used as confounding factor for comparisons between the two tumor spread types in ascites and solid tissue samples (Supplementary Table S2). In Table 1 numbers of significantly differentially expressed sRNAs are shown.

**Table 1:** Numbers of significantly deregulated miRNAs, gene sets, miR sets, gene-miR sets, and piRNAs

Test	Sample subset	Direction	Significant miRNAs		Significant miR sets (QuSAGE)		Significant gene sets (QuSAGE)		Sign. gene-miR sets	Significant piRNAs	
**Miliary vs non-miliary**	**A, S**	**Up in miliary**	401	141	13,768	13,596	0	0	0	61	36
		**Down in miliary**		260		172		0			25
**Miliary vs non-miliary**	**P, M**	**Up in miliary**	1	1	17,298	17,191	10,593	1,709	19,416	37	25
		**Down in miliary**		0		107		8,884			12
**AS vs PM**	**Non-miliary**	**Up in AS**	55	34	0	0	5,518	3,320	0	10	2
		**Down in AS**		21		0		2,198			8
**AS vs PM**	**Miliary**	**Up in AS**	109	28	0	0	1,160	500	49	9	3
		**Down in AS**		81		0		660			6
**S vs A**	**All**	**Up in S**	262	84	n.d.	n.d.^*^	n.d.	n.d.	n.d.	2	0
		**Down in S**		178		n.d.		n.d.			2
**M vs P**	**All**	**Up in M**	0	0	n.d.	n.d.	n.d.	n.d.	n.d.	1	1
		**Down in M**		0		n.d.		n.d.			0

* n.d., not determined.

### Differential gene-miR set analyses

**Figure 2:**
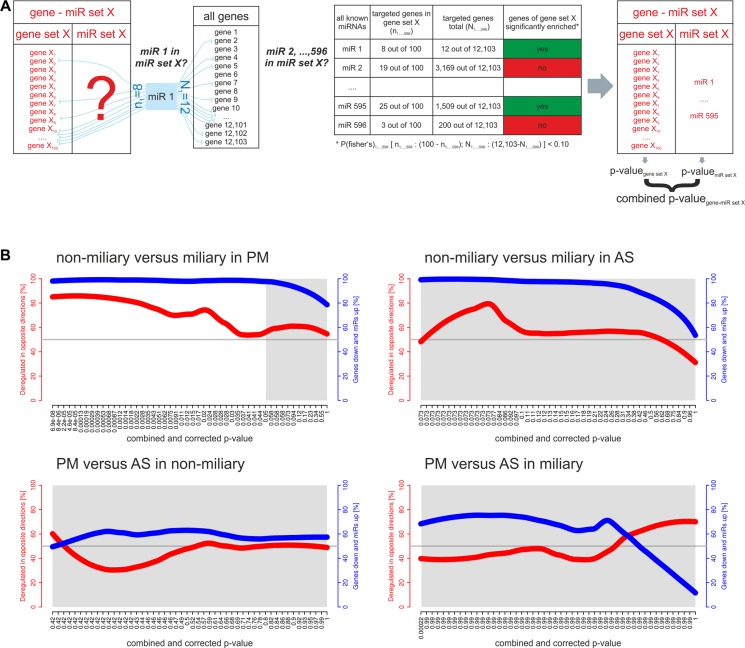
Gene sets and corresponding miR sets (gene-miR sets) (**A**) Assigning miRNAs to targeted genes of gene set (using experimentally verified interactions available for 596 known miRNAs). For each gene set (here exemplarily shown for gene set (X), the fraction (genes of the gene set targeted by each known miRNA/non-targeted genes of the gene set) is compared to the fraction (targeted genes among all genes/non-targeted genes among all genes) by Fisher's exact test. All miRNAs which target significantly more genes (one-sided *p*-value < 0.05) in a gene set than all genes are assigned to the gene set and are referred to as corresponding miR set (both together: gene-miR set). (**B**) Gene-miR sets analyzed together including direction of deregulation. Each gene-miR set is assigned to three values: (i) x-axis: the combined and corrected *p*-value of the gene set and the miR set concerning deregulation in the comparison given in the title. The light grey background represents non-significantly deregulated gene-miR sets (cutoff 0.05). (ii) Left y-axis and red line: percentage of gene-miR sets deregulated in opposite directions. (iii) Right y-axis and blue line: percentage of inversely deregulated gene-miR sets whose gene sets are downregulated and whose miR sets are upregulated in miliary (upper panels) or in AS (lower panels).

We extended the analysis of individual miRNAs to the analysis of sets of miRNAs and performed an analysis similar to gene set enrichment analysis. For this approach we combined the gene sets, which were almost completely comprised of protein-coding genes (*i.e.* 95% of gene sets consist of > 93% protein-coding genes), with their putative miRNA regulators using only experimentally verified miRNAs-target mRNA associations. Figure 2A outlines the gene-miR set analysis approach. Most gene-miR sets comprised > 80% protein-coding genes and < 20% miRNAs (Supplementary Figure S3). Assuming that miRNAs downregulate their target genes, inverse directions of regulation for a gene set and the corresponding miR set would be expected. The following comparisons were made: miliary versus non-miliary in solid tumor tissues (PM) and ascites (AS), and ascites versus solid tumor tissues in miliary and non-miliary. Interestingly, miliary versus non-miliary in solid tumor tissues yielded 19,416 significantly deregulated gene-miR sets, 71.8% of them with gene sets down and the corresponding miR sets upregulated in miliary (Table 1, Figure 2B, upper left). In ascites miliary versus non-miliary no gene-miR set was significantly deregulated (Table 1, Figure 2B, upper right). This implies a more suppressive effect of miRNAs on mRNAs in miliary compared to non-miliary, especially in tumor cells from solid tumor tissues. Moreover, this result proves the validity of our approach of annotating the gene sets with their putative miRNA regulators.

In miliary, 49 gene-miR sets were significantly deregulated between ascites and solid tumor tissues, around 50% of them with gene sets and miR sets deregulated in opposite directions, which is not more than expected by chance. In non-miliary, no gene-miR set was significantly deregulated between ascites and solid tumor tissues (Table 1, Figure 2B, lower panels).

### Competing endogenous (ce) RNA network analysis

**Figure 3:**
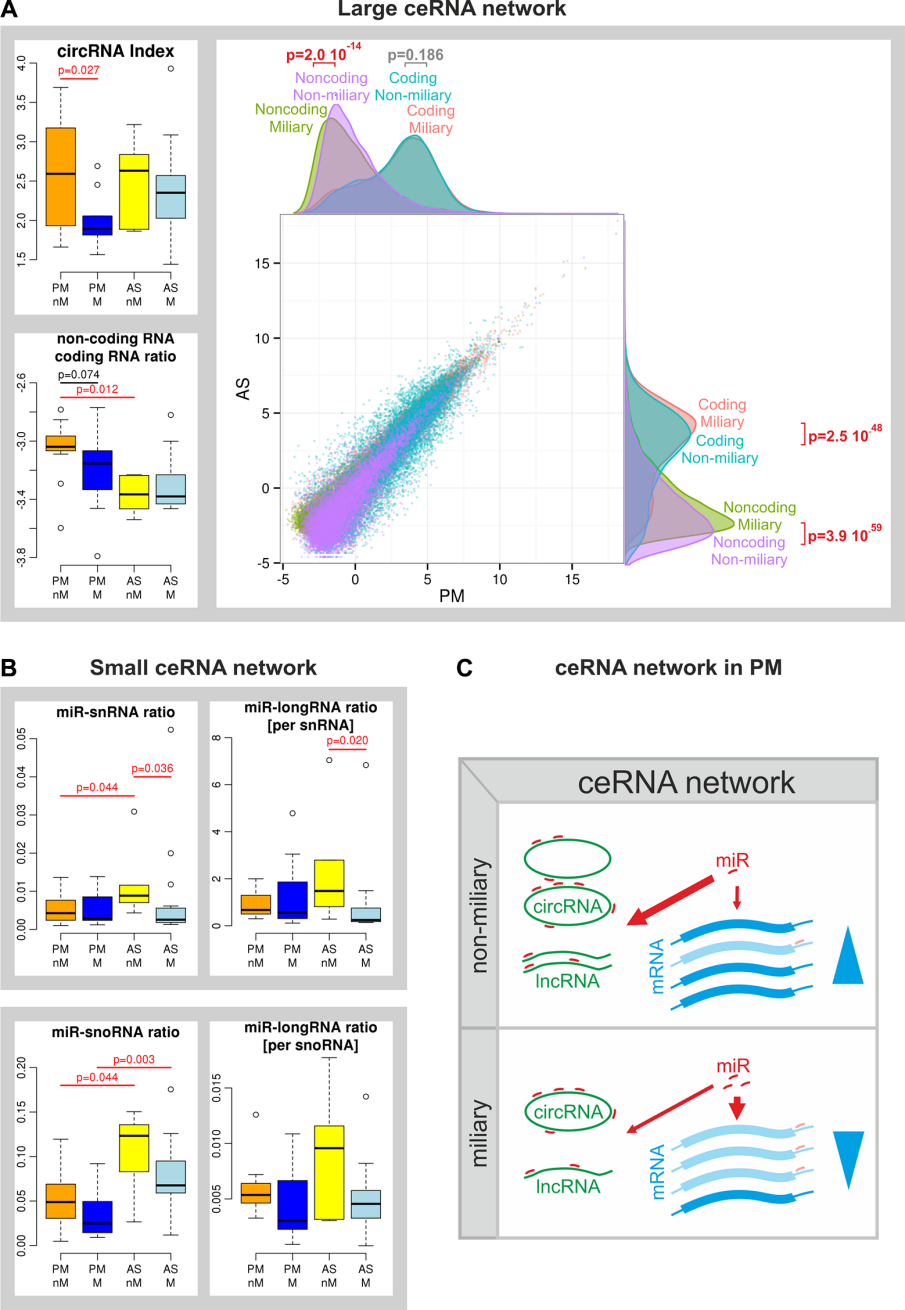
Competing endogenous RNA (ceRNA) network (**A**) Left: boxplots of the circRNA index and of the ratio of linear non-coding to coding RNAs of all samples; right: Scatterplot and corresponding density distribution plots of the mean expression levels of each coding and non-coding RNA averaged over solid (PM) and ascitic (AS) tissue types and over each spread type. (**B**) Left: boxplots of the amount of miRNAs normalized to total small nuclear RNAs (miR-snRNA ratio) and of the ratio of miRNAs to long linear coding and non-coding RNAs both normalized to snRNAs (miRNA-longRNA ratio [per snRNA]); right: boxplot of the amount of miRNAs normalized to total small nucleolar RNAs (miR-snoRNA ratio) and of the ratio of miRNAs to long linear coding and non-coding RNAs both normalized to snoRNAs (miRNA-longRNA ratio [per snoRNA]). Primary ovarian tumor cells (P) and metastatic peritoneal tumor cells (M) as well as ascitic single tumor cells (A) and spheroids (S) were summarized and analyzed for both tumor spread types non-miliary (nM) and miliary (M) separately. *P*-values were calculated with Student's *t*-test. (**C**) Scheme of the ceRNA in solid tumor cells (PM): a strong large ceRNA network resulting in decreased regulatory impact of miRNAs in non-miliary and a reduced large ceRNA network resulting in increased regulatory action of miRNAs in miliary.

The gene-miR set results prompted us to compare the global amount of ncRNAs, constituting the ceRNA network such as miRNAs, circRNAs, lncRNA and coding RNAs. CircRNAs, a recently described species of extremely diverse ncRNAs comprised of mostly coding exons from protein-coding genes and generated by back-splicing events, seem to bind miRNAs (similar to sponges) and prevent them from downregulating their mRNA targets [15, 16]. From rRNA-depleted RNA-sequencing data a circRNA index was calculated (number of total back-splicing events divided by the number of all splicing events) which was significantly decreased in miliary compared to non-miliary PM samples (*p* = 0.027, Figure 3A), indicating a reduced circRNA abundance compared to linear RNA species in miliary compared to non-miliary PM samples. We previously reported a negative correlation of circRNAs with proliferation and proposed a model of passive accumulation of circRNAs in non-proliferating cells [17]. Interestingly, despite the lower circRNA index in miliary PM samples, proliferation (MKI67 expression) was not elevated in miliary compared to non-miliary tumor cells (Supplementary Figure S4). The ratio of the median expression of lncRNAs to coding RNAs showed a trend of reduction in miliary compared to non-miliary PM samples (*p* = 0.074). But a more comprehensive analysis, averaging gene expression values of solid tumor and ascitic tumor tissues for each spread type, showed a significant overall higher expression of lncRNAs in non-miliary compared to miliary solid samples (*p* = 2.0 × 10^−14^) with an unchanged overall expression of coding RNAs (*p* = 0.186). In ascites both, coding and non-coding RNAs, were higher expressed in miliary compared to non-miliary (*p* = 2.5 × 10^−48^ and *p* = 3.9 × 10^−14^, respectively, Figure 3A) but not differentially between non-coding and coding. Total miRNAs (normalized to small nuclear (sn)RNAs or small nucleolar (sno)RNAs) were unchanged in solid tumor tissues of both spread types. Analogously, total piRNAs did not show any difference between the two spread types (Supplementary Figure S5). Finally, the global amount of miRNAs and long linear RNAs (coding and ncRNAs, circRNAs excluded) were compared (normalized both to either snRNAs or snoRNAs). In both approaches the ratio of miRNAs to long RNAs was unaffected (Figure 3B). Taken together, the lower circRNA index and the lower ratio of lncRNAs to coding RNAs in miliary compared to non-miliary PM samples, on the one hand, and the unaffected total abundance of miRNAs and the unaffected ratio of miRNAs to long linear RNAs, on the other hand, suggest that more miRNAs remain unbound by the ceRNA network in miliary PM samples and are therefore available for regulatory functions (Figure 3C).

### Validation of small RNA-sequencing with RT-qPCR

To validate *sRNA-seq* data, 48 miRNAs and piRNAs were selected from the lists of significantly differentially expressed sRNAs and analyzed with RT-qPCR (Supplementary Table S3). Moreover, five sRNAs with very stable expression were included as normalizers (miR-92a-3p, miR-101–3p, miR-103a-3p, miR-106b-5p, and novel piR-n4_chr11_122017273). Additionally, one scrambled sequence was included as negative control, which remained below detection limit in all samples (Cq values > 38). One miRNA (miR-214–3p) was analyzed in duplicates to confirm reproducibility of the technique (R = 0.97). We used the same sRNA preparations subjected to *sRNA-seq* for RT-qPCR (*sRNA-qPCR*). Additionally, solid tumor samples for which matched FFPE tissue blocks were available were subjected to total RNA extraction from macro-dissected tissue sections and also used for RT-qPCR (*FFPE-qPCR*).

All four variants of one miRNA (novel miR-2916), *i.e.* a SNP variant (C/T) and two alternative 3′ ends (with and without a T in the last position), which were predicted using the *sRNA-seq* data, were found to be expressed using four different specific RT-qPCR systems. Only one (miR-3188) of the 18 known miRNAs and four of the 22 newly predicted miRNAs (novel miR-347, −1116, −2364, and −1331) were not reliably expressed (Cq > 38 in > 75% of samples), yielding 94.4% and 81.8% validation rates, respectively. The expression of all five known and three novel piRNAs could be confirmed.

**Figure 4:**
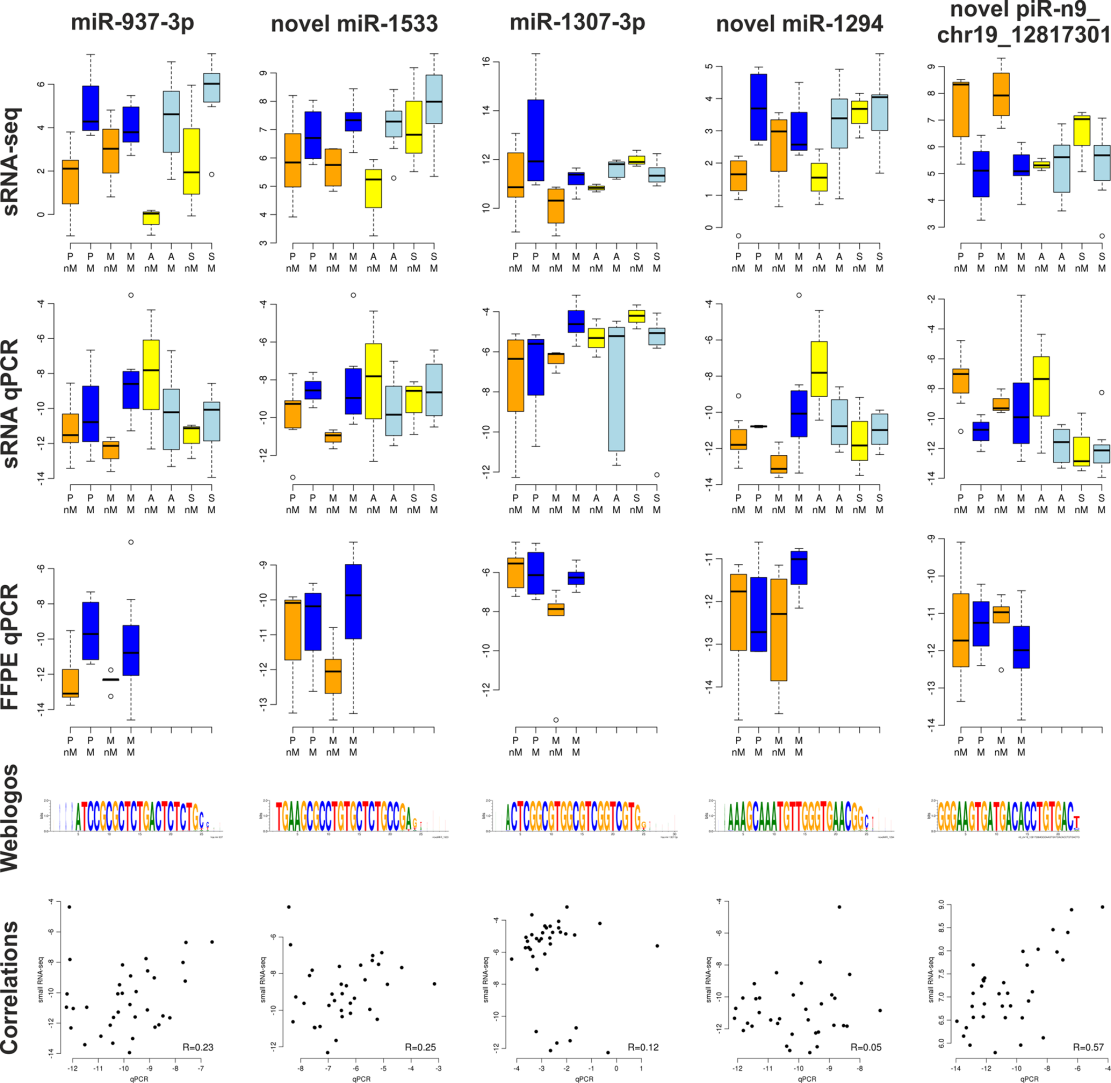
The four most differentially expressed mi/piRNA between miliary (M) and non-miliary (nM) tumor spread Each type of tumor cell sample was analyzed separately: P, primary ovarian tumor; M, metastatic peritoneal tumor; A, ascitic single tumor cells; S, ascitic spheroids. Small RNA sequencing data (sRNA-seq, *n* = 43), qPCR using the same small RNA samples (sRNA-qPCR, *n* = 40), qPCR using total RNA from matched FFPE tissue blocks (FFPE-qPCR, *n* = 23), weblogos showing the sequence diversity of the small RNAs as derived from sRNA-seq (height of each letter indicates the relative frequency of the nucleotide, width of the letter indicates the absolute frequency of this nucleotide in that position), and correlation plots between sRNA-seq and sRNA-qPCR (*n* = 35).

The two different techniques RNA-seq and RT-qPCR were compared using the same RNA samples (*n* = 35). For correlation analyses, only the RNA-seq reads exactly corresponding to the sequence analyzed with RT-qPCR were considered. The median correlation coefficients were 0.23 for miRNAs and 0.37 for piRNAs. We also compared expression of the same 48 sRNAs in enriched tumor cells from fresh tumor tissues (*sRNA-qPCR*) and from macro-dissected FFPE tumor tissue sections (*FFPE-qPCR*). The median correlation coefficients were 0.25 for miRNAs and 0.06 for piRNAs. In Figure 4, the most differentially expressed sRNAs between miliary and non-miliary solid tumor (PM) samples are shown.

### Biological interpretation

We built a high-scoring protein-protein interaction network using putative gene targets (*n* = 897) of the four most differentially expressed miRNAs between miliary and non-miliary (miR-937–3p, miR-1307–3p, novel miR-1533, and novel miR-1294, Supplementary Figure S6). Large RNA-sequencing data of these targets were used to indicate differences in gene expression between miliary and non-miliary solid tumor tissues. Interestingly, TP53 was the hub-gene with most connections. Other hub-genes were minichromosome maintenance complex component 4 *(MCM4*), catenin (cadherin-associated protein) alpha 1 (*CTNNA1*), neural cell adhesion molecule 1 (*NCAM1*), calcium channel voltage-dependent L type alpha-1B and −1D subunit (*CACNA1B*/*1D*), and glial cell line-derived neurotrophic factor (GDNF) family receptor alpha 1 (*GFRA1*).

### Development of a sRNA tumor spread signature

A robust (not model driven) RT-qPCR derived sRNA signature predicting tumor spread behavior was developed using mi- and piRNAs deregulated only between miliary and non-miliary ovarian (P) tumor samples, as FFPE ovarian tumor samples were available from an independent validation cohort (see below). The sRNA signature consisted of three miRNAs which were higher expressed in miliary (miR-760, novel miR-1003, and novel miR-2508) and ten sRNAs which were higher expressed in non-miliary (miR-1254, novel miR-804, novel miR-1628, novel miR-1927, novel miR-2353, novel miR-2916, novel miR-3475, novel miR-3784, piRno_hsa_009295, and novel piRn9_chr19_12817301). This *13 sRNA spread predictor* was calculated for each sample by subtracting the median expression of the ten non-miliary-up sRNAs from the median expression of the three miliary-up sRNA yielding one predictive value. This *13 sRNA spread predictor* has to be interpreted relatively, *i.e.* higher for more miliary and lower for more non-miliary. This predictor separated the training cohort perfectly into non-miliary and miliary (Supplementary Figure S7).

### Validation of the 13 sRNA tumor spread predictor

**Figure 5:**
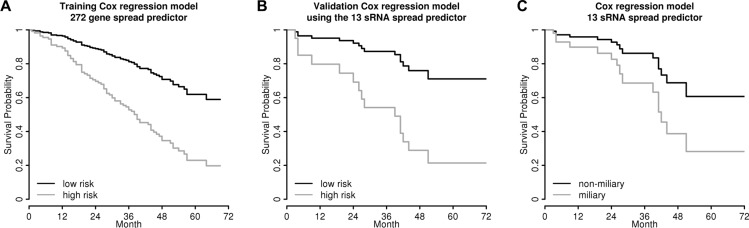
Survival analyses Survival estimates (**A**) of the multiple Cox regression model using the *272 gene spread predictor* (Auer *et al.* 2015) together with age, FIGO stage, residual tumor, and grade in the 165 patients training cohort (hazard ratio (HR) for the *272 gene spread predictor* = 1.74, *p* = 0.028; *cf.* Table 2A), (**B**) of the validation model in the 32 patients validation cohort replacing the *272 gene spread predictor* by the *13 sRNA spread predictor* (comprised of 11 miRNAs and 2 piRNAs) in the Cox regression model of (A) (coef = 1.10 (CI_95_ 0.48–1.71), indicating a positive validation of the training Cox regression model; *cf.* Table 2B), and (**C**) of the independent multiple Cox regression model using the *13 sRNA spread predictor* in the 32 patients cohort, corrected for age and FIGO stage (HR = 2.54, *p* = 0.106; *cf.* Table 2C). Curves represent survival estimates from the corresponding multiple Cox regression models, therefore no censored observations are indicated. Patients were stratified at the median according to high and low risk groups in (A) and (B) and according to miliary and non-miliary with the 13 sRNA spread predictor in (C).

To validate this sRNA signature predictive for tumor spread type and to correlate it with clinical data, we applied it to an independent cohort of 32 HGSOC FIGO III/IV patients with similar clinicopathological characteristics: 24 patients presented with FIGO III and eight with FIGO IV. The median age at diagnosis was 57 years (26–82). Of the 32 patients 15 (46.9%) were already deceased. The median OS time was 50 months (3–91). Their tumor spread types, which were not available from clinicopathologic documentation, were predicted using the *13 sRNA spread predictor*. We used this independent cohort, since the follow-up of the training cohort was too short for survival analysis. The *13 sRNA spread predictor* was correlated to all relevant clinicopathological factors and OS in the validation cohort. It did not correlate with any of the clinicopathological parameters (age, FIGO stage, grade, and residual tumor). As we have already reported an independent negative prognostic impact on OS of the miliary spread type (predicted with a *272 gene spread predictor*) in our previous study [11], we applied this multiple Cox regression model to the validation cohort (Table 2A) replacing the *272 gene spread predictor* with the *13 sRNA spread predictor*. The risk for each patient from the validation cohort was calculated using the Cox regression model from the training cohort with the *13 sRNA spread predictor* value. Subsequentially this risk value was used as a single predictor in the Cox regression model with survival data from the validation cohort. The regression coefficient for this risk value in the Cox model was 1.10 (confidence interval (CI)_95%_ 0.48–1.71, Table 2B), indicating a successful validation of the model, even using two different spread predictors, the *272 gene spread predictor* for the training cohort and the *13 sRNA predictor* for the validation cohort (a successful validation of a Cox regression model is given if the CI_95_ of the regression coefficient of the risk values calculated with the training Cox model using survival data of the validation cohort covers 1). To illustrate the training Cox regression model applied to the test cohort using the *272 gene spread predictor* and to the independent validation cohort using the *13 sRNA spread predictor*, the survival estimates were dichotomized at the median in high and a low risk groups and the survival estimates were plotted for both of them (Figure 5A and 5B).

**Table 2:** Cox regression analyses. A) Training Cox regression model of 165 FIGO II/III/IV FIGO serous ovarian cancer patients using a previously published median dichotomized 272 *gene spread predictor* [11], B) Validation Cox regression model from A) using the 13 small RNA (sRNA) spread predictor and corresponding clinicopathologic parameters from the independent validation cohort of 32 FIGO III/IV high grade serous ovarian cancer patients, C) Cox regression model of the 32 patient cohort from B) dichotomized at the median of the *13 sRNA spread predictor values*

A) *n* = 165, 78 events [11]	Multiple Cox regression		
	Coef	Hazard ratio (HR) (CI_95_)	*p*
**Age (decades)**	**0.38**	**1.46 (1.17–1.81)**	**< 0.001**
**FIGO (IV vs III vs II)**	**1.02**	**2.76 (1.63–4.69)**	**< 0.001**
**Residual tumor (yes vs no)**	**0.70**	**2.02 (1.24–3.30)**	**< 0.001**
Grade (3 vs 2)	0.54	1.72 (0.95–3.10)	0.074
***272 gene spread predictor (median)***	**0.56**	**1.74 (1.06–2.86)**	**0.028**

B) *n* = 32, 15 events	Validation	
	Coef (CI_95_)	HR (CI_95_)
**Validation model**	**1.10 (0.48–1.71)**	**2.99 (1.62–5.51)**

C) *n* = 32, 15 events	Multiple Cox regression		
	Coef	HR (CI_95_)	*p*
**Age (decades)**	**0.84**	**2.31 (1.31–4.06)**	**0.004**
**FIGO (IV vs III)**	**1.13**	**3.09 (1.03–9.30)**	**0.044**
*13 sRNA spread predictor (median)*	0.93	2.54 (0.82–7.84)	0.106

Further, a new Cox model was calculated independently for the validation cohort using the *13 sRNA spread predictor* in order to compare the resulting hazard ratio (HR), *i.e.* 2.54 for miliary (Table 2C), with the HR obtained in the training cohort with the *272 gene spread predictor* [11] (HR = 1.74, CI_95_ 1.06–2.86, Table 2A). Again, the validation HR was within the CI_95_ of the HR in the training cohort, indicating a successful validation. Figure 5C shows the impact of the *13 sRNA spread predictor* on OS in the validation cohort, corrected for age and FIGO stage, with survival estimates stratified at the median of the *13 sRNA predictor* value.

We further validated the prognostic impact of miliary and non-miliary using the prognostic TCGA “high-risk” gene signature [18]: We calculated the TCGA risk-score for all samples of the 23 patients and correlated this score to the (known) spread type. The miliary spread type correlated significantly with a high TCGA high-risk score (*p* = 0.002), further supporting the negative prognostic impact of the miliary spread type.

## DISCUSSION

HGSOC is still a fatal disease. Despite its heterogeneity, most patients are treated with standard therapy regardless of molecular differences. However, recent studies suggest treatment response differences according to molecular characteristics such as mutation status [19] or gene expression signatures [18, 20, 21]. Thus, understanding the molecular basis of HGSOC and peritoneal tumor spread would be of immense importance. To our knowledge, we are the first to perform a detailed rRNA-depleted large and small RNA-seq analysis of matched enriched tumor cell samples from ovary, peritoneum, and ascites in HGSOC.

### Small RNA-sequencing

We found evidence for expression of hundreds of known mi/piRNAs and thousands of novel mi/piRNAs. The comparison of these novel miRNAs to those of two recent studies [13, 14] revealed only 3% overlap, presumably due to high tissue specificity [14, 22] and due to low expression levels of many newly predicted miRNAs (*i.e.* 66% of novel miRNAs with < 100 reads among all samples).

### Differential transcription analysis

Most differentially expressed sRNAs (401 miRNAs and 61 piRNAs) were found between miliary and non-miliary in ascitic tumor cells, whereas very few were found in solid tumor cells (one miRNA and 37 piRNAs). In contrast, almost 20,000 gene-miR sets (out of 24,991) were significantly deregulated between miliary and non-miliary solid tumor cells (> 70% with downregulated gene sets and upregulated miR sets in miliary), whereas in ascitic tumor cells no gene-miR set was deregulated. This finding is in line with our previous study where > 200 genes, but only 29 gene sets were deregulated between miliary and non-miliary in ascitic tumor cells, while only two genes, but > 6,000 gene sets (out of 10,294) were deregulated between miliary and non-miliary solid tumor cells (most of the genes and gene sets downregulated in miliary) [11].

We also performed a general analysis assessing whole functional RNA groups such as coding RNAs, lncRNAs, circRNAs, and sRNAs (miRNAs and piRNAs). Interestingly, we found evidence for an reduced ceRNA network in miliary compared to non-miliary solid tumors, indicated by reduced levels of circRNAs and lncRNAs compared to linear protein-coding RNAs (Figure 3A) in miliary compared to non-miliary solid tumor samples. The reduced level of circRNA could not be explained by a higher proliferation rate [17] (MKI67 expression was even lower), therefore another (active) mechanism causing lower levels of circRNAs and lncRNAs has to be suggested. This finding together with the unaffected level of miRNAs (also compared to coding and long non-coding RNAs, Figure 3B) indicates more unbound miRNAs available for regulation in miliary compared to non-miliary PM samples (Figure 3C).

Analyzing gene-miR sets and the complete ceRNA network constituents, we extended the analysis of individual gene or sRNA expressions to a more global view on transcription and transcription regulation. This new view on expression data presumably allows finding individually smaller, but in total possibly larger effects, which are often overseen in analyses focusing only on single gene/miRNA deregulations. Furthermore, the effect of the compromised ceRNA network (*i.e.* increasing the regulatory action of unbound miRNAs) in miliary could be even underestimated, since miRNAs mainly regulate protein expression by inhibiting mRNA translation (in case of imperfect sequence complementarity with the target), not by inducing mRNA degradation (in case of perfect complementarity). Summarizing, a picture of a weaker ceRNA network and consequently more unsponged miRNAs, probably reducing protein levels substantially, in the miliary compared to the non-miliary solid tumors emerges (Figure 3C).

### Validation of sRNA-seq with RT-qPCR

Using RT-qPCR, an overall existence validation rate of 87.5% for miRNAs and 100% for piRNAs was achieved, grossly confirming the sRNA-seq data. However, the median correlation coefficients between sequencing and RT-qPCR data were poor (< 0.4). Reasons for this could be that the used pre-designed systems for known miRNAs did not always correspond to the most prevalent sRNA-seq sequences (isomiRs) in our samples. Further, we could not adequately cover undefined 3′-ends using the Exiqon RT-qPCR technique (not allowing the design of different 3′-ends). The median correlation coefficients between *sRNA-qPCR* and *FFPE-qPCR* was even worse (< 0.3), presumably caused by differences in the tumor microenvironment (immune, stromal, or endothelial cells and/or exosomal sRNAs): For *sRNA-seq* and *sRNA-qPCR* EpCAM positive tumor cells were enriched removing components of the microenvironment and exosomes, whereas macro-dissected FFPE tissues were not enriched for tumor cells. Indeed, differential miRNA 3′-modifications and a differential distribution of small noncoding RNA families between cells and exosomes have been reported [23].

### Biological interpretation

High scoring protein-protein interaction sub-network analysis using published and predicted targets of the four most differentially expressed miRNAs between miliary and non-miliary revealed several genes involved in fundamental processes well-known for cancer initiation and progression. The most important hub-gene was the well-known tumor suppressor gene *TP53* whose mutation (in 96% of HGSOC patients) is one of the first known molecular events in the development of HGSOC [18]. Further, several genes at central nodes in the sub-network have cell-cell and cell-matrix adhesion functions and were described in numerous human cancer entities: *CTNNA1* [24, 25], *NCAM1* [26, 27], and *GFRA1* [28, 29]. *MCM4* which was also at a central position in the network is essential for DNA replication and has already been described in other human cancer entities [30–32].

### Tumor spread signature and validation

Our aim was to define a robust sRNA signature to predict tumor spread behavior from FFPE material. We selected sRNAs from RT-qPCR data most characteristic for the two spread types yielding a *13 sRNA tumor spread predictor.* We used this signature to predict the tumor spread behavior of an independent validation cohort of 32 patients with long follow-up data in order to validate the negative prognostic impact of the miliary tumor spread type recently reported by us [11]. In this previous study we built a Cox regression model using a *272 gene spread predictor*. By using the *13 sRNA spread predictor* instead of the *272 gene spread predictor* we could successfully validate this Cox regression model showing the negative impact on OS of the miliary spread type (corrected HR = 2.54, *cf.* initial corrected HR = 3.77 [11]). We further confirmed the negative prognostic impact of miliary by showing a positive association of the miliary spread type with “high-risk” according to a signature published by the TCGA consortium [18].

Moreover, this *13 sRNA spread predictor* can be used to classify patients with non-determinable tumor spread (due to very widespread peritoneal involvement) in clinical routine, which could influence the treatment strategy. The relatively low number of sRNAs comprising the predictor and its applicability for FFPE material would make this predictor a useful, powerful, and robust tool for clinical implementation.

Local peritoneal tumor spread is a special characteristic for and constitutes high-risk in HGSOC. Unlike the majority of cancer entities characterized by ‘conventional’ distant metastasis, in HGSOC the ‘direct’ peritoneal route for metastasizing circumventing the lymph and blood circulatory system is of great importance. The results of our previous study [11] together with those of the current one suggests that processes of epithelial to mesenchymal transition (EMT) seem more important in non-miliary compared to miliary tumor spread. It could even be possible that the few bulky peritoneal implants in patients with non-miliary spread type are real distant metastases, i.e. metastasizing via EMT-MET through the blood (or lymph) system. In contrast, tumor cells of the miliary spread type seem to be ideally adapted for survival in anaerobic ascites and for direct implantation on the peritoneal surface due to globally decreased gene expression activity (downregulation of numerous coding genes by enhanced miRNA regulation due to a reduced ceRNA network) and other metabolic adaptations. This is also in accordance with the counterintuitive impact of high CCNE1 expression and Ki-67 abundance on overall survival, *i.e.* both predictive for favorable overall survival, recently observed by us [33, 34]. Together, the less active cell state (defined by reduced protein production) and more epithelial phenotype of miliary tumor cells could be (one of) the causes for the stronger peritoneal involvement and thus worse outcome in this patient group. The revealed differences between the two spread types could be the starting point for exploring new molecular therapeutic targets for patients with miliary tumor spread. Here, we provide a sRNA signature to diagnose these patients even in very early or advanced stages.

We were the first to subject tumor cell enriched tumor samples from ovarian and peritoneal tumors and from ascites of ovarian cancer patients to a combined analysis of the rRNA-depleted large and small (*i.e.* the complete) transcriptome. Many new miRNAs and piRNAs were predicted and exemplarily validated. The negative impact on survival of the miliary spread type was validated in an independent cohort using a 13 sRNA expression signature. In miliary compared to non-miliary solid tumors, only few individual miRNAs (and large RNAs [11]) were deregulated but numerous gene-miR sets. A global RNA analysis revealed a putative reduction of the ceRNA network presumably causing an increased miRNA regulation in miliary compared to non-miliary solid tumors. Knowing about these differences and having the possibility to classify patients according to spread type (using a 13 small RNA signature) is a first step to develop new specific therapeutic strategies for both spread types.

## MATERIALS AND METHODS

For a more detailed description of materials and methods see Supplementary Materials and Methods.

### Patient information, sample preparation, and RNA extraction (see Supplementary Materials and Methods)

#### Library synthesis and sequencing

NEBNext Multiplex Small RNA Library Prep Set for Illumina (New England Biolabs, Ipswich, MA, USA) was used for library preparation according to the manufacturer's instructions. Sequencing was performed with 50 bp single end reads on a HiSeq 2000 (Illumina, San Diego, CA, USA).

#### cDNA synthesis and RT-qPCR

cDNA synthesis was performed with the miRCURY LNA Universal RT microRNA PCR Starter kit (Exiqon, Vedbaek, Denmark). RT-qPCR was performed using microRNA single assays and ready to use Pick and mix plates (Exiqon).

#### Bioinformatical and statistical analyses

The genome version used for all analyses was HG19. Reads were trimmed and all 13–37 nt long and complete reads were retained (UEA sRNA workbench V3.0_L [35]). All 18–23 nt reads which were supported by at least five reads among all samples were used for miRNA prediction using miRDeep* (v32 [36]) and default parameters. All predicted miRNAs were annotated with known human miRNAs (miRBase v20 [12]). Identical and similar reads (isomiRs) were collapsed. piRNAs were predicted from all reads with proTRAC v2.0.1 [37] and intersected with known piRNAs (NCBI 37, *n* = 23,437) from piRNABank (http://pirnabank.ibab.ac.in/) and accordingly annotated. piRNA loci yielding the same or very similar mature piRNAs were collapsed. Read numbers were cyclic loess normalized and are given as Log_2_((read count+0.5)/millions of total counts). For statistical analysis only mi/piRNAs with > 100 supporting reads among all samples were included. Statistical analyses were performed with R (v3.1.2).

We built gene-miR sets starting with gene sets from the GSEA (Broad Institute, version 4.0) and provided in R-package GeoDE. Each gene set was annotated with their putative miRNA regulators by assigning known miRNAs to their putative targets (experimentally verified miRNA-target interactions, miRTarBase, release 4.5, Figure 2A). For ceRNA network analysis, the overall amounts of ncRNAs such as miRNAs, circRNAs, and lncRNAs and coding RNAs [11] were analyzed. The high-scoring protein-protein interaction network between published and predicted targets of differentially expressed miRNAs was built with R-package dnet (v. 1.0.7) as described in [11].

A robust predictive signature for the miliary and non-miliary spread type was defined using *sRNA-qPCR* data [38] with R-package CellMix. Univariate and multiple Cox proportional hazards regression analyses were used to evaluate the marginal and adjusted association of our developed sRNA tumor spread predictor and commonly used clinicopathological factors [39].

Two sided *p*-values < 0.05 or FDR < 5% were regarded as significant in all analyses (if not stated otherwise).

## SUPPLEMENTARY MATERIALS FIGURES AND TABLES






