# Supplementary material for: Small RNAs and the competing endogenous RNA network in high grade serous ovarian cancer tumor spread
Source: Oncotarget. 2016 May 9;7(26):39640–53. doi: 10.18632/oncotarget.9243 (PMC5129959; doi:10.18632/oncotarget.9243)
Supplement: Supplementary file 1 [file oncotarget-07-39640-s001.pdf]

Small RNAs and the competing endogenous RNA network in high grade serous ovarian cancer tumor spread

Supplementary Materials

MATERIALS AND METHODS

Patient information

23 chemo-naïve HGSOC patients were consecutively enrolled between March 2012 and May 2013 at the Medical University of Vienna. Fresh tumor tissues from ovarian and peritoneal origin as well as whole ascites were collected from these patients. Further retrospective 32 chemo-naïve HGSOC patients were used as validation cohort. All patients signed an informed consent. The institutional ethical review board approved this study (IRB-no: 793/2011).

Peritoneal tumor spread was assessed during surgery and classified into miliary and non-miliary as described [1]. Only those patients whose tumor spread type could be determined as miliary or non-miliary were enrolled in the study (for very late stages this classification was often not possible). Relevant clinicopathological factors, histology, grade, FIGO stage, and lymph node status were assessed by gynecological pathologists. TP53 mutations were assessed by a functional assay (FASAY) and confirmed by Sanger sequencing.

Sample preparation and RNA extraction

Fresh tumor tissues of ovarian and peritoneal origin and ascites collected during cytoreductive surgery were treated as described before [1]. In brief, solid tumor tissues were minced and enzymatically digested to obtain single cell suspensions. Ascites was subsequently filtered using 150 µm, 30 µm, and 20 µm mesh size filters. The fraction between 150 µm and 30 µm was termed spheroids; the flow through of the 20 µm filter was termed ascites single cells. The single cell fractions from the solid tumors and ascites were enriched for EpCAM+ cells with magnetic beads.

Library synthesis and sequencing

NEBNext Multiplex Small RNA Library Prep Set for Illumina (New England Biolabs, Ipswich, Massachusetts, USA) was used for library preparation on an epMotion 5075 automated pipetting system (Eppendorf, Hamburg, Germany) according to the manufacturer's instructions with following specifications: starting material of 30 ng small RNA (< 200 nt), twelve PCR cycles in the final PCR reaction, two AMPure bead (Beckman Coulter, Pasadena, CA, USA) clean-ups after ligation and two AMPure bead clean-ups after PCR. Libraries were quality controlled and the ratio of adapter dimer to correct library size was calculated with Agilent 2100 Bioanalyzer (Agilent Technologies, USA). Library quantification was performed with a digital droplet PCR (ddPCR) system (Q × 100™ Droplet Digital™ PCR System, BioRad, Hercules, CA, USA) and the ddPCR Library Quantification Kit for Illumina TruSeq (BioRad). The ratio adapter dimer to library with insert and the absolute quantification values were used for equimolar pooling of twelve libraries per lane. Sequencing was performed with 50 bp single end reads on a HiSeq 2000 (Illumina, San Diego, CA, USA).

RNA extraction from FFPE tissues

Total RNA was extracted from FFPE tissues from two to eight 10-nm sections. Tissue sections were stained with hematoxylin according to standard procedures. Tumor tissue was macroscopically dissected. In case of very small tumor cell islets, tissues were laser micro-dissected using the MMI CellCut system (Molecular Machines & Industries, Zurich, Switzerland). Total RNA was prepared using the FFPE allPrep kit (Qiagen) with xylene for deparaffinization on a QIAcube (Qiagen).

cDNA synthesis and RT-qPCR

For cDNA synthesis with the miRCURY LNA Universal RT microRNA PCR Starter kit (Exiqon, Vedbaek, Denmark) following RNA amounts were used: 2 ng of enriched small RNAs from fresh tissues; 10–15 ng of total RNA isolated from FFPE tissue sections. RT-qPCR using microRNA PCR single assays (Exiqon) was performed according to the manufacturers' recommendations with 1:50 diluted cDNA. qPCR using ready to use Pick and mix plates (Exiqon) was performed with 1:75 diluted cDNA. For one miRNA (novel miR-2916) four PCR systems were designed, covering a SNP variant (C/T) and two 3′ ends (with and without a T at the last position) (Supplementary Table S3). A scrambled sequence with a GC-content

similar to the median GC content of the other selected miRNA sequences (65%) tested not to align to any known human sequences was included as negative control (GUCAAGCCAUUGCCUCUCGGGC).

Bioinformatical and statistical analyses

Reads were trimmed using the adaptor sequence (AGATCGGAAGAGCACACGTCT) and all complete reads (*i.e.* followed by an adaptor) between 13 and 37 bases were retained (UEA sRNA workbench V3.0_L [2]). All reads between 18 and 23 nt were used for miRNA prediction using miRDeep* (v32 [3]) and default parameters. All predicted miRNAs were annotated with known human miRNAs (miRBase v20 [4]). For miRNA quantification, identical reads were summarized and miRNAs with similar sequences (isomiRs) were collapsed tolerating only length variations, but no mismatches or insertions/deletions. miRNA loci were visualized among chromosomes with Idiographica [5].

piRNAs were predicted from all reads with proTRAC v2.0.1 [6] and intersected with known piRNAs (NCBI 37, n = 23,437) from piRNABank (http://pirnabank.ibab.ac.in/) and accordingly annotated. piRNA loci yielding the same mature piRNAs were collapsed, only length variations were tolerated.

Continuous, normally distributed variables were compared between two groups by Student's t-test. The association of categorical variables was evaluated by Fisher's exact tests.

Differential sRNA expression analysis was performed with R-package limma with its function voom and estimating sample weights [7].

We built gene-miR sets starting with gene sets from the GSEA (Broad Institute, version 4.0) and provided in R-package GeoDE. Each gene set was annotated with their putative miRNA regulators by assigning known miRNAs to their putative targets for which experimentally verified miRNA-target interactions have been reported (miRTarBase, release 4.5). miRNAs enriched for targets in a particular gene set (one-sided p-values < 0.05; Fisher's exact test) were assigned to this particular gene set (Figure 2A). Quantitative Set Analysis for Gene Expression (QuSAGE [8]) analysis was used to determine differential expression of each gene set with RNA-sequencing data and the corresponding miR set with small RNA-sequencing data. For significance analysis both p-values were combined by the Fisher's method and corrected for multiple testing by the Benjamini & Hochberg method.

For ceRNA network analysis, the overall amounts of ncRNAs such as miRNAs, circRNAs, and lncRNAs and coding RNAs [1] were analyzed and compared between AS, PM, miliary, and non-miliary samples. A circRNA index was calculated by dividing the number of reads associated to circRNAs (back-spliced) with the number of reads associated to all splicing events [9]. lncRNA reads and protein-coding reads were summarized from RNA-seq data according to the used gene model. Proliferation of samples was assessed by the MKI67 expression. miRNAs and piRNAs were normalized to small nuclear RNAs (snRNAs) or small nucleolar RNAs (snoRNAs), *i.e.* reads from the *sRNA-seq* data assigned to snRNAs or snoRNAs. Long linear RNAs were calculated by summing up coding and ncRNAs, but excluding circRNAs, and normalized to snRNAs or snoRNAs (reads from the *large RNA-seq* data assigned to snRNAs or snoRNAs).

To evaluate the RT-qPCR data, the expression values (Cq values) were normalized to the geometric mean Cq values of the five normalizer sRNAs: miR-92a-3p, miR-101-3p, miR-103a-3p, miR-106b-5p, and novel piR-n4_chr11_122017273. Differences in cDNA synthesis and PCR efficiency between samples were corrected with an internal spike-in control (UniSp6). Finally the normalized values were multiplied by -1 to be interpretable as $\log_2$-expression values. Reliably expressed was defined as Cq < 38 in > 75% of samples. Correlation analyses were assessed by Spearman's rank correlation coefficient.

dnet R-package (v1.0.7) and information of targets of the four most differentially expressed miRNAs between miliary and non-miliary samples in solid tumors and the corresponding $\log_2$ fold changes were used for construction of the high-scoring protein-protein sub-network on the background of the STRING v9 database. For known miRNAs, targets were taken from the Exiqon database (miRSearch V3.0); for newly predicted miRNAs, targets were predicted with TargetScan.

A robust (not model driven) predictive small RNA signature was defined by the CellMix R-package (v1.6 [10]) using the method from Abbas *et al.* and *sRNA-qPCR* data from primary ovarian tumor samples [11]. Finally, the median expression of non-miliary-up mi/piRNAs was subtracted from the median expression of miliary-up mi/piRNAs yielding in one predictive value (sRNA spread predictor) for each sample.

Univariate and multiple Cox proportional hazards regression analyses were used to evaluate the marginal and adjusted association of our developed sRNA tumor spread predictor and commonly used clinicopathological factors [12]. To validate the sRNA spread predictor, we applied it to a Cox model built with a previously published spread predictor consisting of 272 gene expression values (*272 gene spread predictor*) [1]. The information for Yoshihara's molecular subclass [13] and the peritoneal carcinomatosis status for the validation cohort was not available, therefore these factors were omitted. Only FIGO stage, grade, residual tumor, and age were included as clinicopathologic covariates in the model. Survival estimates were plotted from both cohorts, training and validation, dichotomizing the final model at the median. To assess the corrected impact of the *13 sRNA spread predictor* on OS in the validation cohort, survival curves of the Cox regression model were shown, dichotomized by the sRNA predictor at the median. Additionally, we calculated the risk score for each sample of the 23 patients of our original cohort according to the TCGA signature

using R-package *genefu* v2.2.0 to validate the prognostic effect of the spread type [14]. A linear regression model to predict this risk score using the parameters miliary versus non-miliary, tissue, and patient was performed.

REFERENCES

1. Auer K, Bachmayr-Heyda A, Aust S et al. Peritoneal tumor spread in serous ovarian cancer-epithelial mesenchymal status and outcome. Oncotarget. 2015; 6:17261–17275.
2. Stocks MB, Moxon S, Mapleson D et al. The UEA sRNA workbench: a suite of tools for analysing and visualizing next generation sequencing microRNA and small RNA datasets. Bioinformatics. 2012; 28:2059–2061.
3. An J, Lai J, Lehman ML, Nelson CC. miRDeep*: an integrated application tool for miRNA identification from RNA sequencing data. Nucleic Acids Res. 2013; 41:727–737.
4. Kozomara A, Griffiths-Jones S. miRBase: annotating high confidence microRNAs using deep sequencing data. Nucleic Acids Res. 2014; 42:D68–73.
5. Kin T, Ono Y. Idiographica: a general-purpose web application to build idiograms on-demand for human, mouse and rat. Bioinformatics. 2007; 23:2945–2946.
6. Rosenkranz D, Zischler H. proTRAC—a software for probabilistic piRNA cluster detection, visualization and analysis. BMC Bioinformatics. 2012; 13:5.
7. Liu R, Holik AZ, Su S et al. Why weight? Modelling sample and observational level variability improves power in RNA-seq analyses. Nucleic Acids Res. 2015; 43:e97.
8. Yaari G, Bolen CR, Thakar J, Kleinstein SH. Quantitative set analysis for gene expression: a method to quantify gene set differential expression including gene-gene correlations. Nucleic Acids Res. 2013; 41:e170.
9. Bachmayr-Heyda A, Reiner AT, Auer K et al. Correlation of circular RNA abundance with proliferation—exemplified with colorectal and ovarian cancer, idiopathic lung fibrosis, and normal human tissues. Sci Rep. 2015; 5:8057.
10. Gaujoux R, Seoighe C. CellMix: a comprehensive toolbox for gene expression deconvolution. Bioinformatics. 2013; 29:2211–2212.
11. Abbas AR, Wolslegel K, Seshasayee D et al. Deconvolution of blood microarray data identifies cellular activation patterns in systemic lupus erythematosus. PLoS One. 2009; 4:e6098.
12. Cox DR. Regression Models and Life-Tables. Journal of the Royal Statistical Society Series B-Statistical Methodology. 1972; 34:187-&.
13. Pils D, Hager G, Tong D et al. Validating the impact of a molecular subtype in ovarian cancer on outcomes: A study of the OVCAD Consortium. Cancer Sci. 2012.
14. TCGA. Integrated genomic analyses of ovarian carcinoma. Nature. 2011; 474:609–615.

Supplementary Table S1: Clinicopathological parameters of patients enrolled in the study and performed analyses

Patient	Age	FIGO	Grade	Lymph nodes[1]	Spread[2]	Ascites (ml)	Residual Tumor[3]	TP53 mutation	sRNA-seq[4]	sRNA-qPCR[4]	FFPE-qPCR[4]
5	80	IIIC	G2	pN1	1	< 500	1	yes	M		M
6	70	IIIC	G2	pNX	2	> 500	1	yes	A		
8	53	IIIC	G2	pN1	0	0	0	yes	A, P	A, P	P
9	62	IIIC	G2	pNX	2	> 500	1	yes	A		
12	55	IIIC	G3	pN1	0	> 500	0	yes	P	P	P
13	60	IIIC	G3	pN1	2	> 500	1	yes	A, S	A, S, M	
16	64	IIIC	G3	pNX	2	> 500	1	yes	S, P, M	A, S, P, M	P, M
21	68	IIIC	G3	pNX	2	> 500	0	yes	A, S, M	A, S, M	M
24	50	IIIB	G2	pNX	2	> 500	1	yes	A, S, P	A, S, P	P
25	50	IIIC	G3	pN1	2	> 500	0	yes	A, S, P, M	A, S, P, M	P, M
27	66	IIIA	G3	pN0	1	0	0	yes	P	P	P
28	57	IIIC	G3	pNX	2	< 500	0	yes	A, S, M	A, S, M	M
29	53	IIIC	G3	pN0	1	< 500	0	no	M	M	M
30	41	IIIC	G3	pN0	1	< 500	0	yes	M		M
35	56	IIIB	G3	pNX	2	0	0	yes	M	M	M
39	50	IIIC	G3	pN0	2	0	0	yes	P, M	M	P, M
41	49	IIC	G3	pN0	0	> 500	0	yes	A, S	A, S, P	
42	49	IIIB	G3	pN0	1	> 500	0	yes	A, S, P	A, S	P
53	48	IIIC	G3	pN1	1	0	0	yes	P, M	P, M	P,M
54	34	IV	G3	pNX	2	> 500	1	yes	A, S, M	S, M	M
55	81	IIA	G3	pN0	0	< 500	0	yes	P	P	P
58	66	IIIC	G3	pNX	1	0	0	yes		P, M	M
62	54	IIIC	G3	pN1	1	< 500	0	yes	S, P	S	P

[1]pN0, no regional lymph node metastasis; pN1, regional lymph node metastasis; pNX, regional lymph nodes not assessed.
[2]0, no peritoneal metastases; 1, non-miliary; 2, miliary.
[3]0, no residual tumor; 1, macroscopic residual tumor.
[4]A, ascites single cells; S, ascites aggregated cells (“spheroids”); P, solid ovarian tumor mass (“primary tumor”); M, solid peritoneal tumor mass (“metastasis”).

Supplementary Table S2: List of differentially expressed miRNAs and piRNAs between spread types and tissue types (provided as own Excel-file). See Supplementary_Table_S2

Supplementary Table S3: Known and novel predicted miRNAs and piRNAs analyzed in this study. See Supplementary_Table_S3

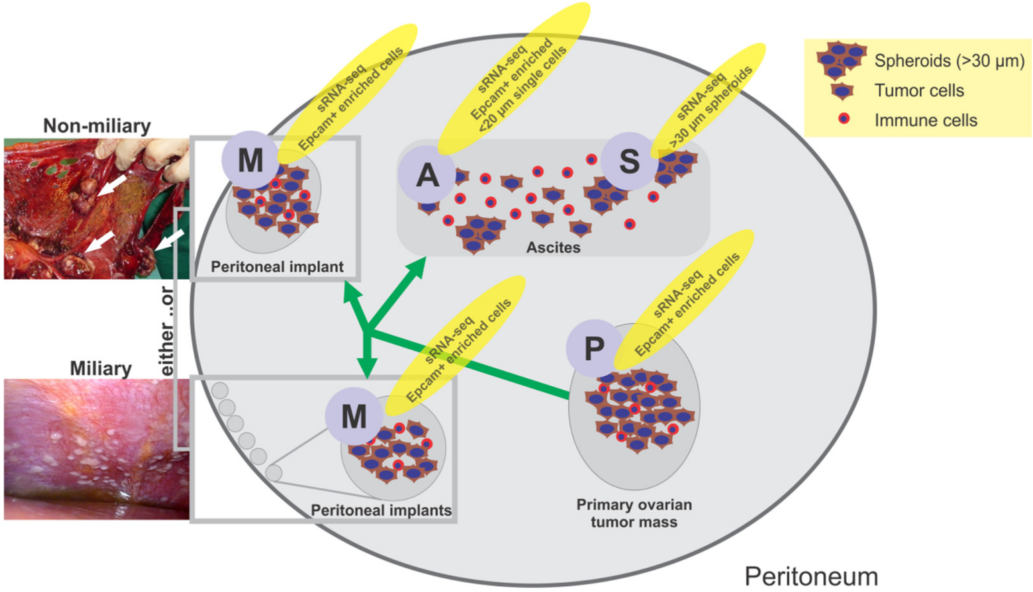

Supplementary Figure S1: Study design. Primary ovarian tumor mass (P), metastatic peritoneal implant (M), and ascites were collected from patients with either miliary (numerous, small implants) or non-miliary (fewer bigger implants, marked with white arrows) peritoneal tumor spread. Solid tumors were digested enzymatically. Ascites was filtered into ascitic single cells (A) < 20 µm and ascitic spheroids (S) > 30 µm. Single cell fractions from solid tumors and ascites were enriched for EpCAM+ cells. These four tumor cell sample types were subjected to small RNA isolation, library synthesis, and small RNA sequencing (sRNA-seq).

Cluster 1

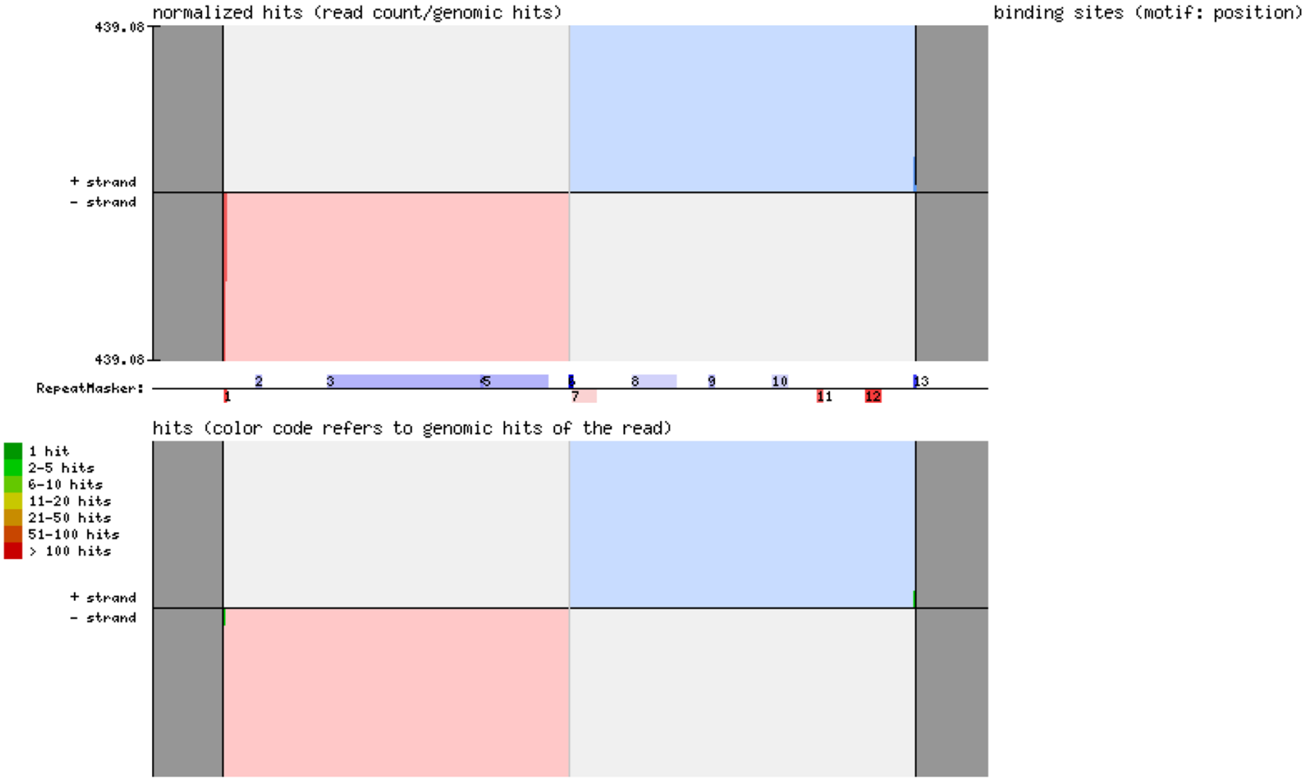

Location: chr1
Coordinates: 147781065-147790886
Size [bp]: 9822
Hits (absolute, non-identical): 72
Hits (normalized): 550.702438404083
Hits (normalized) per kb: 56.068258847901
Normalized hits with 1T: 82.9%
Normalized hits with 10A: 11.5%
Normalized hits with length 24-32 nt: 98.3%
Normalized hits on the main strand(s): 100%
Predicted directionality: bi:minus-plus (split between 147781078 and 147790847)

RepeatMasker annotation:

100-98% identity
<98-95% identity
<95-90% identity
<90-85% identity
<85-80% identity
<80-75% identity
<75-70% identity
<70% identity

1: tRNA-Glu-GAA 147781030-147781101
2: tRNA-Lys-AAG 147781527-147781590
3: L1MD1 147782546-147784710
4: MLT1B 147784711-147784760
5: L1MD1 147784763-147785658
6: (CA)n 147785955-147786005
7: MLT2B4 147786006-147786347
8: MER67B 147786856-147787475
9: L1M5 147787947-147788023
10: L1ME3G 147788848-147789051
11: tRNA-Asn-AAC 147789483-147789554
12: AluJb 147790161-147790384
13: tRNA-His-CAY_ 147790852-147790918

Cluster 2

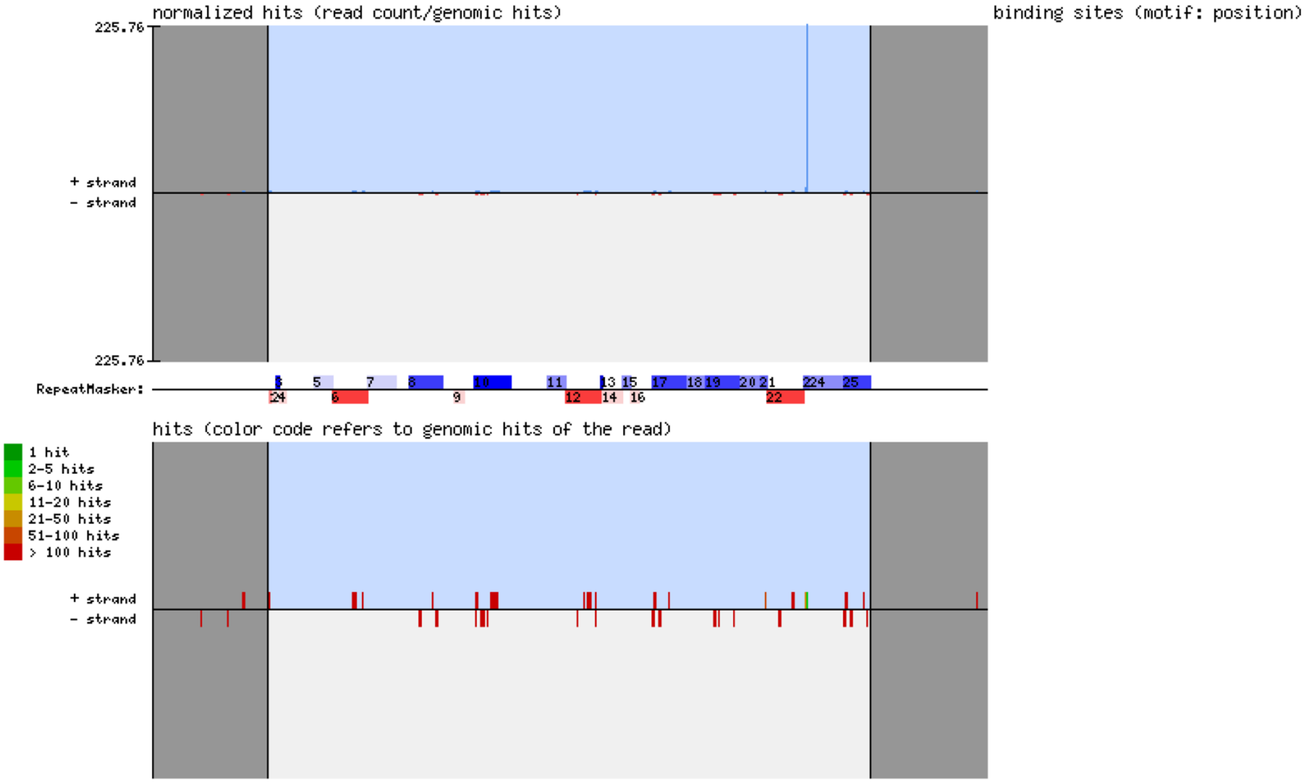

Location: chr5
Coordinates: 154080637-154085807
Size [bp]: 5171
Hits (absolute, non-identical): 357
Hits (normalized): 226.459577594144
Hits (normalized) per kb: 43.7941554040116
Normalized hits with 1T: 15.5%
Normalized hits with 10A: 83%
Normalized hits with length 24-32 nt: 100%
Normalized hits on the main strand(s): 100%
Predicted directionality: mono:plus

RepeatMasker annotation:

100-98% identity
<98-95% identity
<95-90% identity
<90-85% identity
<85-80% identity
<80-75% identity
<75-70% identity
<70% identity

1: AluJo 154080547-154080665
2: MER96B 154080668-154080684
3: (TTTG)n 154080685-154080715
4: MER96B 154080716-154080770
5: MLT1K 154081022-154081178
6: AluSx3 154081179-154081480
7: MLT1K 154081481-154081727
8: AluSq2 154081834-154082131
9: MIR 154082220-154082316
10: AluSx3 154082399-154082707
11: AluJo 154083032-154083180
12: AluSx1 154083183-154083480
13: AT_rich 154083481-154083503
14: L2a 154083504-154083668
15: MER75A 154083669-154083743
16: L2a 154083744-154083815
17: AluSq2 154083925-154084227
18: L1PA15 154084228-154084395
19: AluSx 154084396-154084694
20: L1PA15 154084695-154084858
21: HY1 154084859-154084921
22: AluSx1 154084922-154085233
23: HY1 154085234-154085293
24: L1PA15 154085294-154085573
25: AluSx1 154085574-154085855

Cluster 3

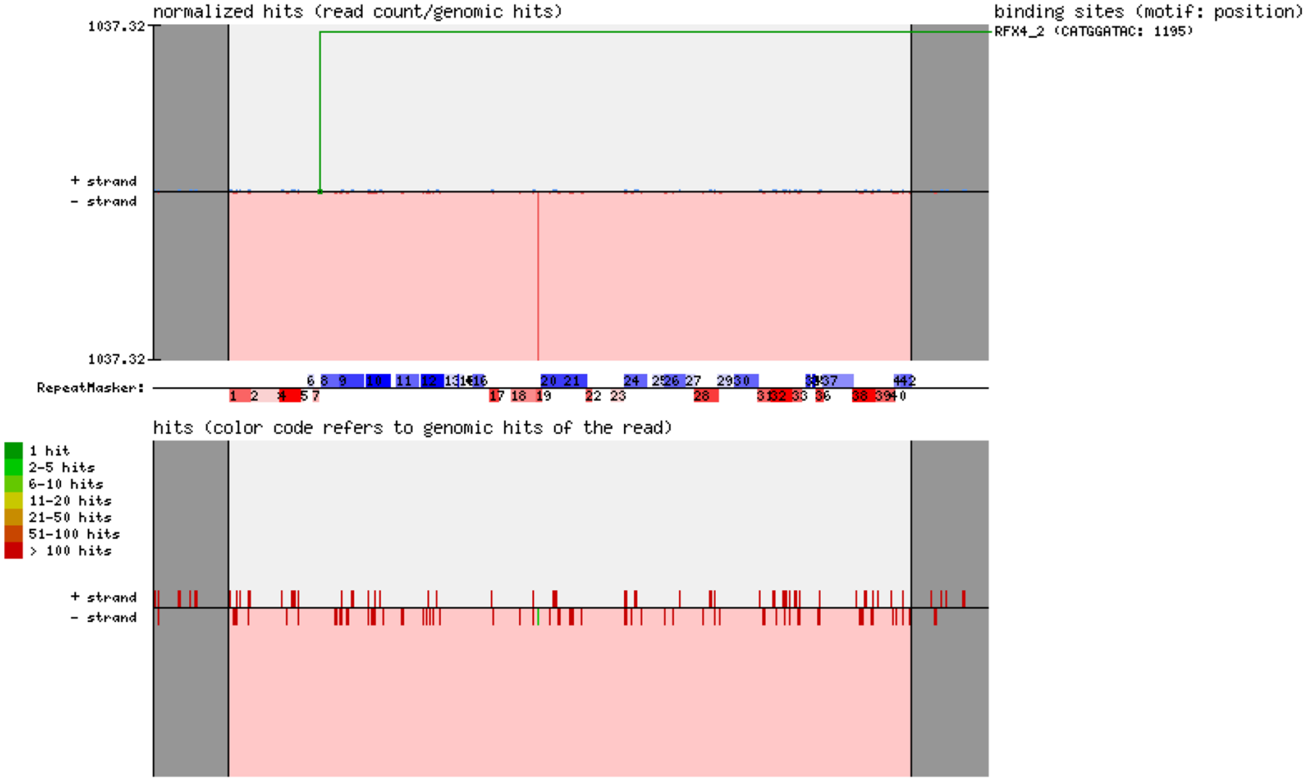

Location: chr7
Coordinates: 75374921-75383961
Size [bp]: 9041
Hits (absolute, non-identical): 890
Hits (normalized): 1039.22030073644
Hits (normalized) per kb: 114.945282682938
Normalized hits with 1T: 27.1%
Normalized hits with 10A: 71.8%
Normalized hits with length 24-32 nt: 100%
Normalized hits on the main strand(s): 100%
Predicted directionality: mono:minus

RepeatMasker annotation:

100-98% identity
<98-95% identity
<95-90% identity
<90-85% identity
<85-80% identity
<80-75% identity
<75-70% identity
<70% identity

1: AluY 75374894-75375218
2: LTR47A 75375219-75375561
3: L1MC4a 75375562-75375592
4: AluSg 75375593-75375869
5: L1MC4a 75375870-75375902
6: MIRc 75375952-75376023
7: L2a 75376033-75376088
8: AluJo 75376148-75376378
9: AluSz 75376381-75376688
10: AluSx 75376745-75377037
11: AluSx 75377138-75377419
12: AluY 75377467-75377770
13: L1MC4a 75377775-75377940
14: (CA)n 75377941-75377979
15: L1MC4a 75377980-75378019
16: FLAM_A 75378147-75378271
17: AluJb 75378371-75378487
18: AluJo 75378667-75378986
19: HY1 75378998-75379049
20: AluSz 75379050-75379352
21: AluSx1 75379353-75379652
22: HY1 75379653-75379724
23: L2a 75379985-75380143
24: AluSq2 75380156-75380436
25: L1ME3 75380543-75380671
26: AluSz 75380683-75380969
27: AluJb 75380970-75381092
28: AluSq2 75381093-75381388
29: AluJb 75381389-75381560
30: AluJr 75381617-75381917
31: AluSx 75381920-75382105
32: AluY 75382106-75382383
33: AluSx 75382384-75382505
34: (TTTCC)n 75382569-75382604
35: (TTA)n 75382660-75382682
36: AluJo 75382698-75382783
37: LTR59 75382784-75383179
38: AluSc5 75383183-75383482
39: AluJo 75383483-75383698
40: AluSx 75383699-75383742
41: AluJb 75383743-75383826
42: AluSx 75383828-75384124

Cluster 4

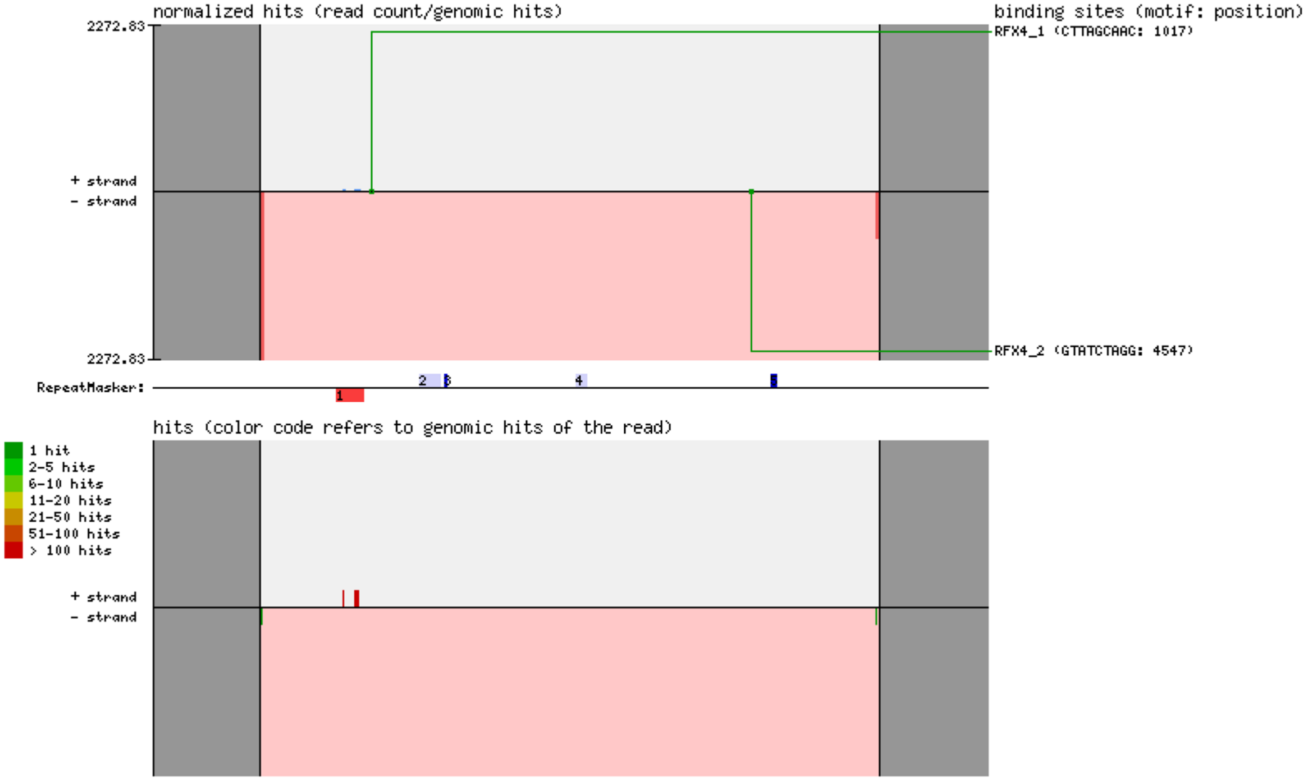

Location: chr11
Coordinates: 122017273-122023006
Size [bp]: 5734
Hits (absolute, non-identical): 23
Hits (normalized): 2893.83333333333
Hits (normalized) per kb: 504.679688408325
Normalized hits with 1T: 76.5%
Normalized hits with 10A: 96.7%
Normalized hits with length 24-32 nt: 100%
Normalized hits on the main strand(s): 100%
Predicted directionality: mono:minus

RepeatMasker annotation:

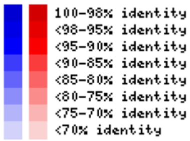

1: AluSg 122017969-122018214
2: L2c 122018744-122018920
3: AT_rich 122018965-122018985
4: L2c 122020189-122020284
5: (CA)n 122022002-122022044

Cluster 5

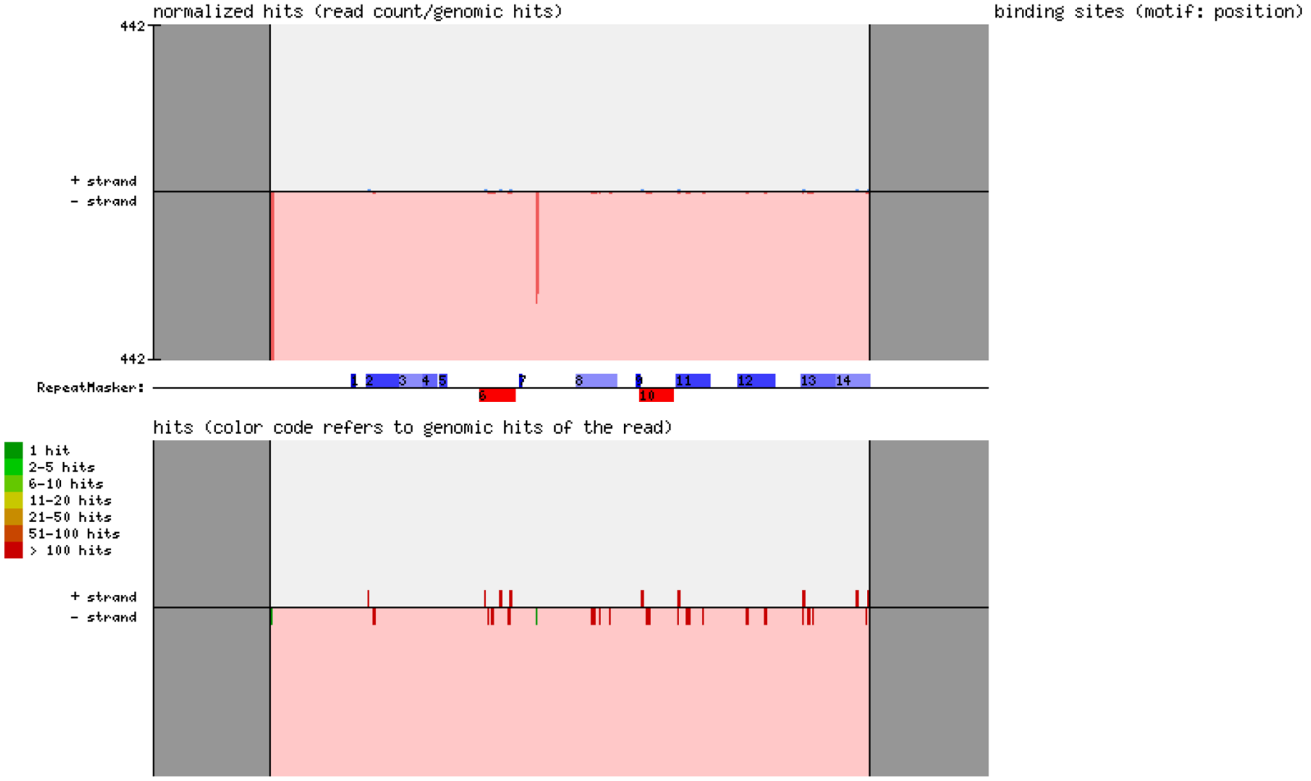

Location: chr12
Coordinates: 49048268-49053392
Size [bp]: 5125
Hits (absolute, non-identical): 234
Hits (normalized): 974.060493284598
Hits (normalized) per kb: 190.060584055531
Normalized hits with 1T: 64.3%
Normalized hits with 10A: 64.4%
Normalized hits with length 24-32 nt: 72.8%
Normalized hits on the main strand(s): 100%
Predicted directionality: mono:minus

RepeatMasker annotation:

100-98% identity
<98-95% identity
<95-90% identity
<90-85% identity
<85-80% identity
<80-75% identity
<75-70% identity
<70% identity

1: AT_rich 49048959-49048979
2: AluJb 49049087-49049365
3: L1MB4 49049369-49049560
4: AluJr 49049562-49049679
5: L1MB3 49049712-49049761
6: AluSc8 49050047-49050354
7: (A)n 49050394-49050413
8: AluSc 49050884-49051228
9: (TG)n 49051388-49051417
10: AluSq2 49051418-49051699
11: AluSz 49051736-49052016
12: AluSx 49052257-49052568
13: AluSz6 49052796-49053087
14: AluJo 49053101-49053417

Cluster 6

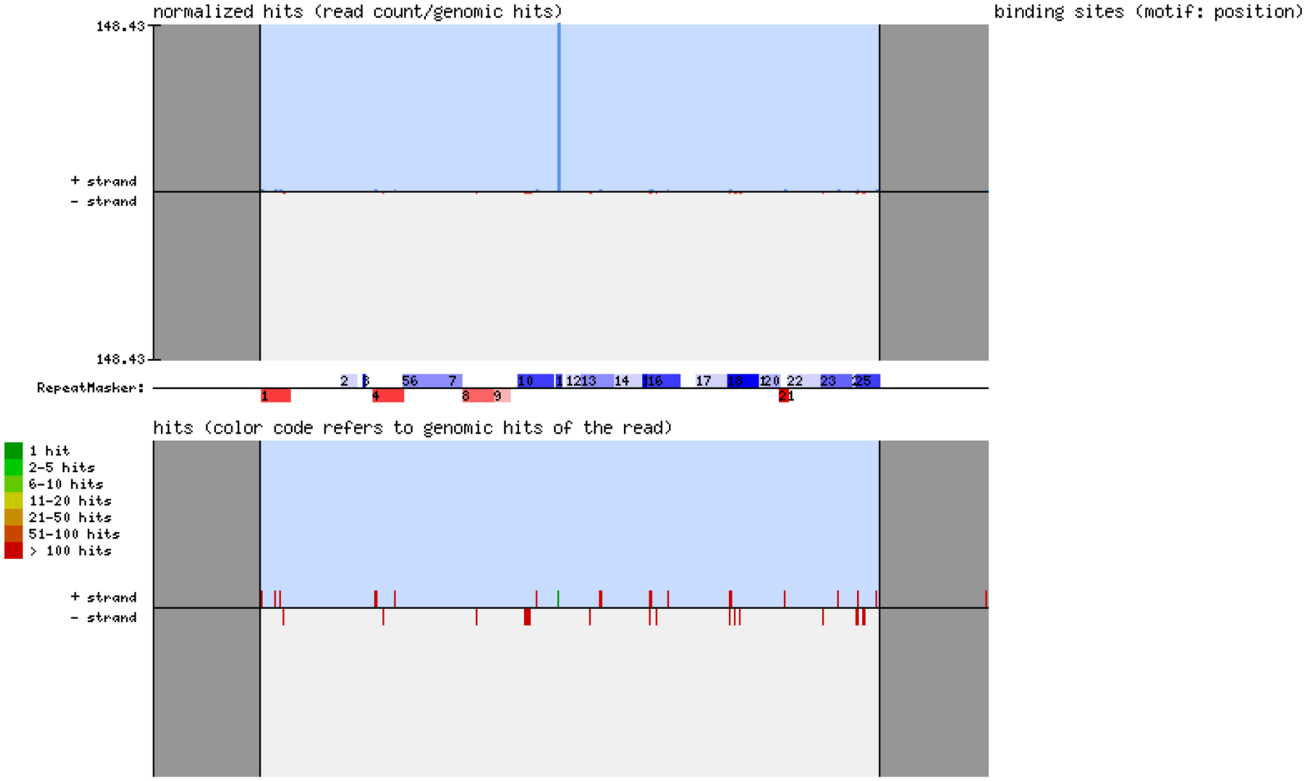

Location: chr14
Coordinates: 64243352-64249135
Size [bp]: 5784
Hits (absolute, non-identical): 192
Hits (normalized): 148.455428150026
Hits (normalized) per kb: 25.6665677991055
Normalized hits with 1T: 27.3%
Normalized hits with 10A: 72.5%
Normalized hits with length 24-32 nt: 100%
Normalized hits on the main strand(s): 100%
Predicted directionality: mono:plus

RepeatMasker annotation:

100-98% identity
<98-95% identity
<95-90% identity
<90-85% identity
<85-80% identity
<80-75% identity
<75-70% identity
<70% identity

1: AluSz 64243306-64243618
2: FRAM 64244107-64244249
3: AT_rich 64244304-64244327
4: AluJr4 64244400-64244685
5: L1MA9 64244686-64244756
6: THE1D 64244757-64245119
7: L1MA9 64245120-64245218
8: AluJr 64245243-64245532
9: AluJr 64245541-64245677
10: AluSq 64245756-64246078
11: HY1 64246118-64246165
12: L1M4 64246198-64246349
13: AluSz 64246350-64246654
14: L1M4 64246655-64246922
15: L1ME2z 64246918-64246969
16: AluSx1 64246970-64247267
17: L1ME2z 64247425-64247714
18: AluSp 64247715-64248015
19: L1ME2z 64248016-64248084
20: L1MC4 64248060-64248195
21: AluYc 64248197-64248273
22: L1MC4 64248274-64248578
23: AluJo 64248579-64248879
24: L1MC4 64248880-64248909
25: AluSq2 64248910-64249207

Cluster 7

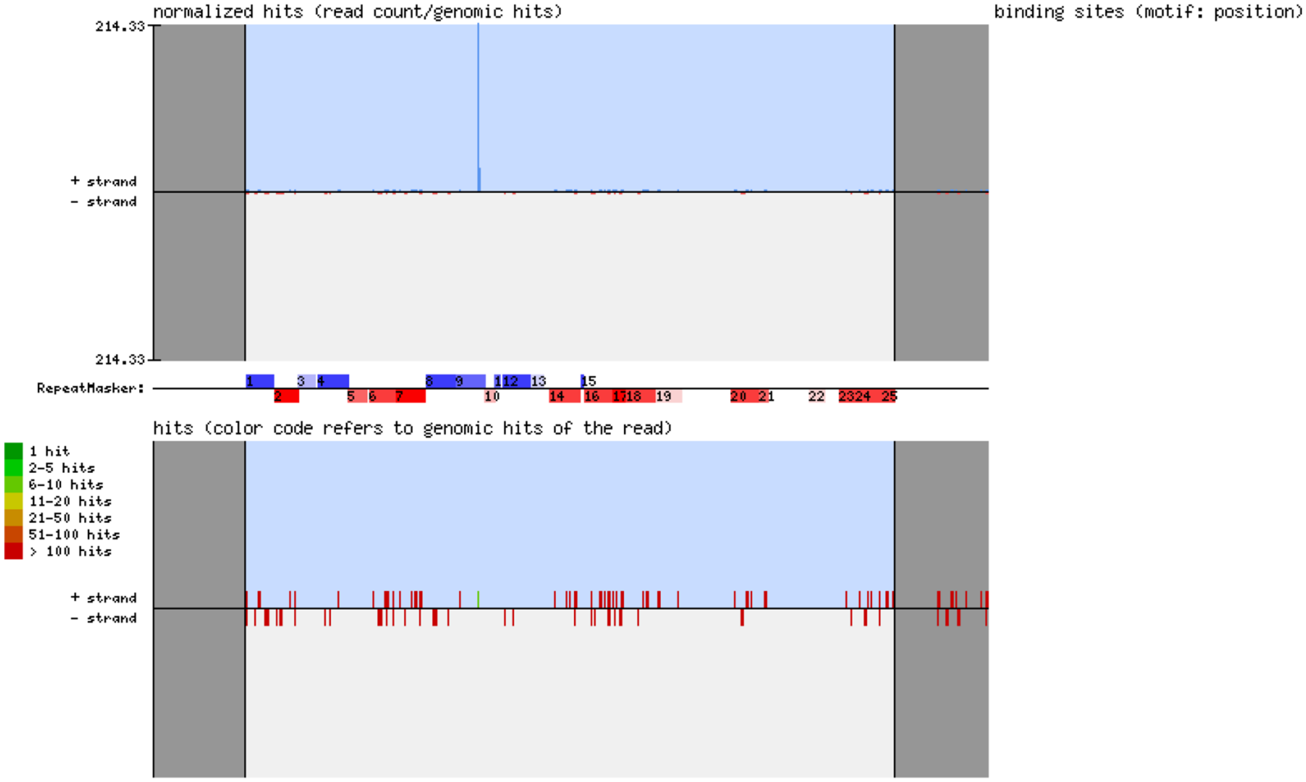

Location: chr16
Coordinates: 2060907-2067911
Size [bp]: 7005
Hits (absolute, non-identical): 695
Hits (normalized): 242.070143483612
Hits (normalized) per kb: 34.5567656650409
Normalized hits with 1T: 5.6%
Normalized hits with 10A: 70.3%
Normalized hits with length 24-32 nt: 94.8%
Normalized hits on the main strand(s): 99.9%
Predicted directionality: mono:plus

RepeatMasker annotation:

100-98% identity
<98-95% identity
<95-90% identity
<90-85% identity
<85-80% identity
<80-75% identity
<75-70% identity
<70% identity

1: AluSx 2060896-2061195
2: AluY 2061203-2061461
3: AluSx 2061462-2061642
4: AluY 2061685-2062000
5: MLT1D 2062003-2062199
6: AluSc8 2062239-2062533
7: AluSp 2062534-2062825
8: AluSx1 2062850-2063149
9: 7SLRNA 2063182-2063477
10: MLT1D 2063486-2063591
11: MER20 2063592-2063640
12: AluSx1 2063681-2063991
13: HAL1 2063992-2064117
14: AluSx 2064192-2064502
15: (T)n 2064519-2064550
16: AluSx1 2064553-2064862
17: AluYk11 2064865-2064998
18: AluSz 2065020-2065326
19: AluJb 2065336-2065599
20: AluSx1 2066148-2066449
21: AluSz6 2066460-2066550
22: MIR 2066997-2067152
23: AluSx 2067317-2067492
24: AluSg 2067493-2067790
25: AluSx 2067791-2067925

Cluster 8

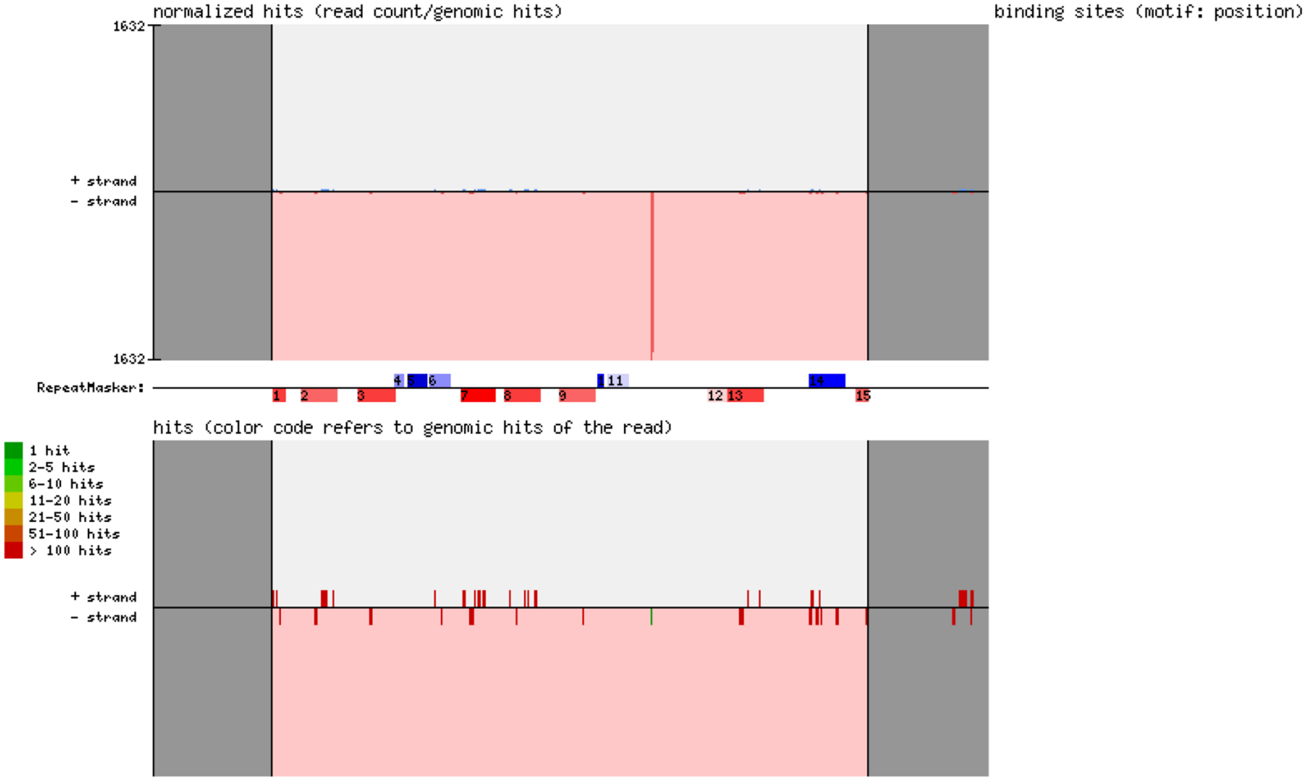

Location: chr17
Coordinates: 57211948-57216980
Size [bp]: 5033
Hits (absolute, non-identical): 327
Hits (normalized): 1632.06343710599
Hits (normalized) per kb: 324.272488993839
Normalized hits with 1T: 99%
Normalized hits with 10A: 99%
Normalized hits with length 24-32 nt: 100%
Normalized hits on the main strand(s): 100%
Predicted directionality: mono:minus

RepeatMasker annotation:

100-98% identity
<98-95% identity
<95-90% identity
<90-85% identity
<85-80% identity
<80-75% identity
<75-70% identity
<70% identity

1: AluSx3 57211917-57212056
2: AluSz 57212193-57212493
3: AluSx1 57212672-57212976
4: MER63D 57212977-57213046
5: AT_rich 57213095-57213244
6: FAM 57213272-57213448
7: AluSq2 57213540-57213824
8: AluSc 57213913-57214207
9: AluJo 57214375-57214674
10: AT_rich 57214699-57214733
11: MIR3 57214784-57214951
12: Tigger4a 57215627-57215783
13: AluSx3 57215789-57216092
14: AluY 57216477-57216783
15: FLAM_C 57216881-57217010

Cluster 9

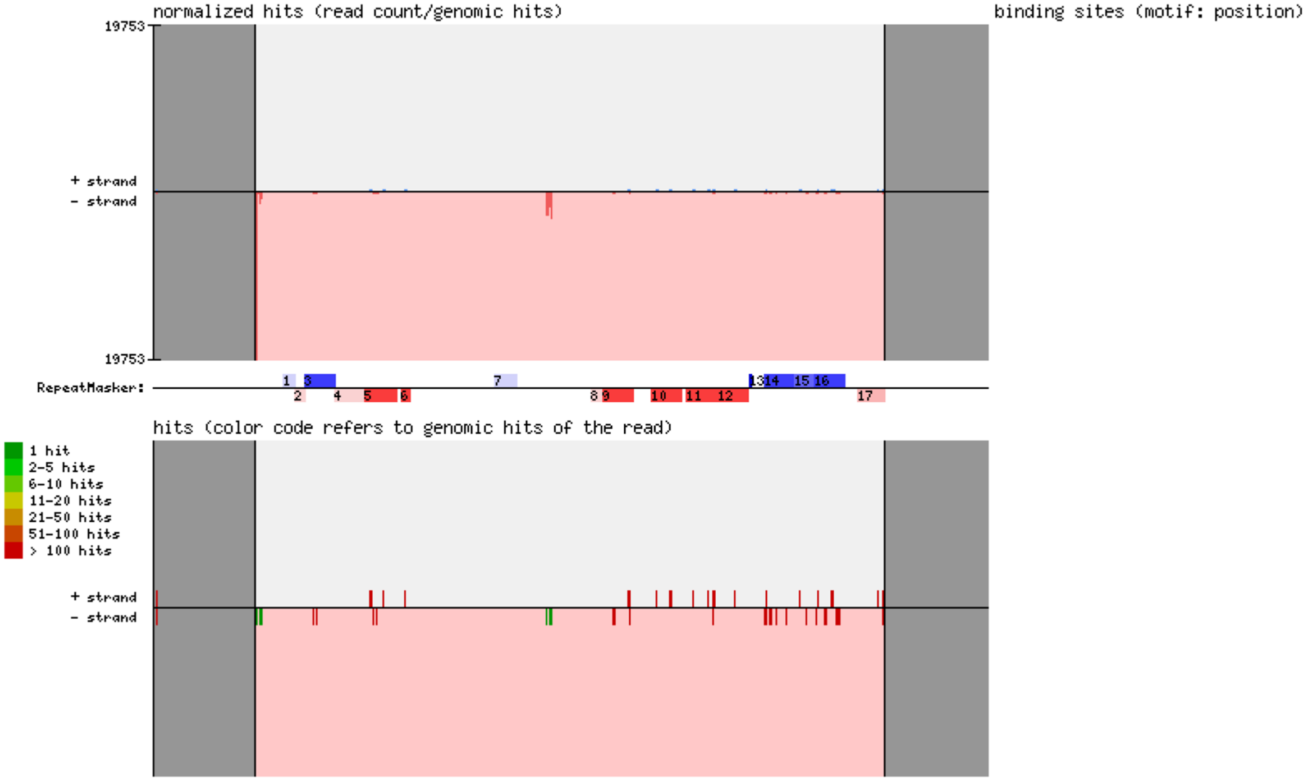

Location: chr19
Coordinates: 12814412-12820582
Size [bp]: 6171
Hits (absolute, non-identical): 470
Hits (normalized): 28235.5294115097
Hits (normalized) per kb: 4575.51926940686
Normalized hits with 1T: 60.8%
Normalized hits with 10A: 52.4%
Normalized hits with length 24-32 nt: 89.3%
Normalized hits on the main strand(s): 100%
Predicted directionality: mono:minus

RepeatMasker annotation:

100-98% identity
<98-95% identity
<95-90% identity
<90-85% identity
<85-80% identity
<80-75% identity
<75-70% identity
<70% identity

1: MIRb 12814671-12814793
2: MER2 12814798-12814897
3: AluSz6 12814898-12815188
4: MER2 12815189-12815455
5: AluSx1 12815484-12815781
6: FLAM_C 12815830-12815919
7: L2b 12816761-12816967
8: MIRb 12817694-12817808
9: AluSc8 12817813-12818110
10: AluSx1 12818292-12818581
11: AluSx 12818625-12818935
12: AluSz6 12818947-12819243
13: AT_rich 12819246-12819267
14: AluSq2 12819399-12819698
15: AluSq2 12819699-12819876
16: AluSc 12819894-12820192
17: AluJo 12820309-12820596

Cluster 10

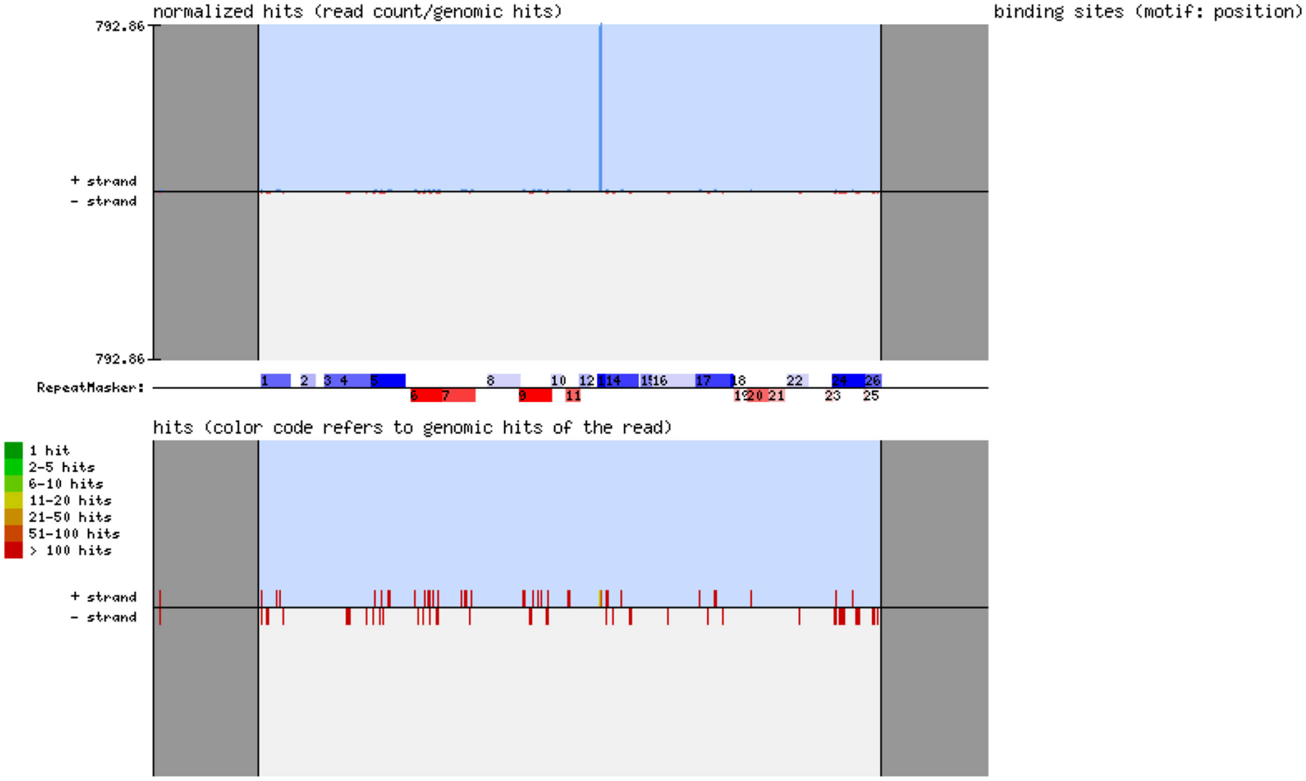

Location: chr19
Coordinates: 46359341-46365155
Size [bp]: 5815
Hits (absolute, non-identical): 557
Hits (normalized): 792.928963991917
Hits (normalized) per kb: 136.359237143924
Normalized hits with 1T: 97.4%
Normalized hits with 10A: 3.2%
Normalized hits with length 24-32 nt: 100%
Normalized hits on the main strand(s): 100%
Predicted directionality: mono:plus

RepeatMasker annotation:

100-98% identity
<98-95% identity
<95-90% identity
<90-85% identity
<85-80% identity
<80-75% identity
<75-70% identity
<70% identity

1: AluSx 46359331-46359607
2: MIR 46359722-46359845
3: AluJr 46359949-46360090
4: AluSx 46360093-46360384
5: AluY 46360385-46360692
6: AluY 46360754-46361048
7: AluSp 46361057-46361356
8: L2a 46361472-46361776
9: AluSp 46361777-46362072
10: L2a 46362073-46362164
11: AluJo 46362204-46362331
12: L2a 46362333-46362429
13: HY1 46362506-46362561
14: AluSz6 46362579-46362886
15: AluJr 46362905-46362999
16: AluJr 46363018-46363406
17: AluSq2 46363432-46363741
18: (GA)n 46363753-46363779
19: MIR 46363780-46363840
20: AluJb 46363906-46364108
21: MIRc 46364115-46364256
22: AluSz6 46364292-46364470
23: L1MC4a 46364647-46364716
24: AluSg 46364717-46365008
25: L1MC4a 46365009-46365020
26: AluSz 46365021-46365313

Cluster 11

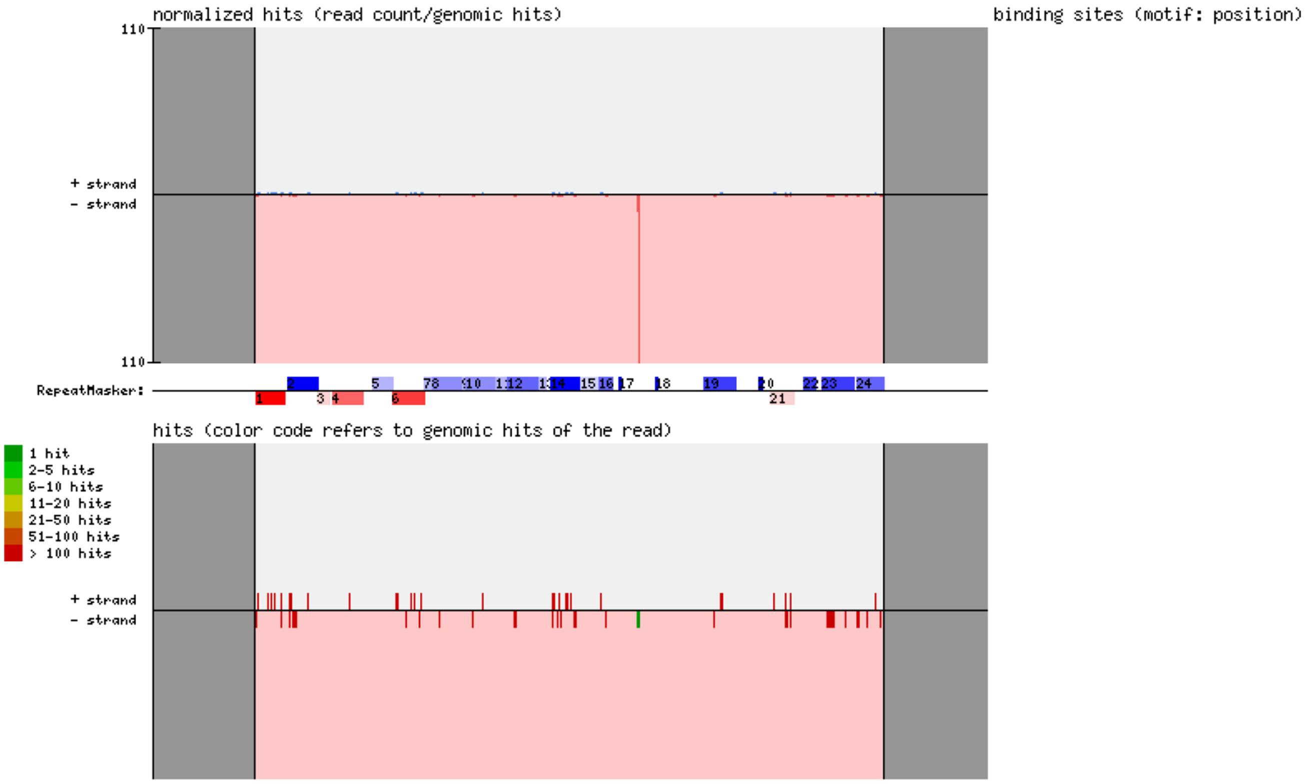

Location: chr20
Coordinates: 35541426-35547519
Size [bp]: 6094
Hits (absolute, non-identical): 344
Hits (normalized): 110.078066360374
Hits (normalized) per kb: 18.0633518805995
Normalized hits with 1T: 0%
Normalized hits with 10A: 99.9%
Normalized hits with length 24-32 nt: 100%
Normalized hits on the main strand(s): 100%
Predicted directionality: mono:minus

RepeatMasker annotation:

100-98% identity
<98-95% identity
<95-90% identity
<90-85% identity
<85-80% identity
<80-75% identity
<75-70% identity
<70% identity

1: AluY 35541390-35541699
2: AluSx1 35541725-35542023
3: MIR 35542026-35542138
4: AluSz6 35542160-35542456
5: Tigger2a 35542548-35542748
6: AluSx1 35542749-35543062
7: Tigger2a 35543063-35543131
8: AluJr 35543132-35543432
9: Tigger2a 35543433-35543464
10: AluJr 35543465-35543756
11: Tigger2a 35543757-35543857
12: AluSz 35543858-35544164
13: Tigger2a 35544165-35544234
14: AluY 35544281-35544574
15: MIR 35544581-35544752
16: FLAM_A 35544754-35544880
17: AT_rich 35544946-35544969
18: AT_rich 35545301-35545321
19: AluSg4 35545775-35546080
20: AT_rich 35546314-35546335
21: AluJb 35546423-35546653
22: FLAM_C 35546740-35546867
23: AluSx 35546920-35547234
24: AluJb 35547258-35547547

Supplementary Figure S2: piRNA clusters. piRNAs were predicted with proTRAC v.2.0.1.

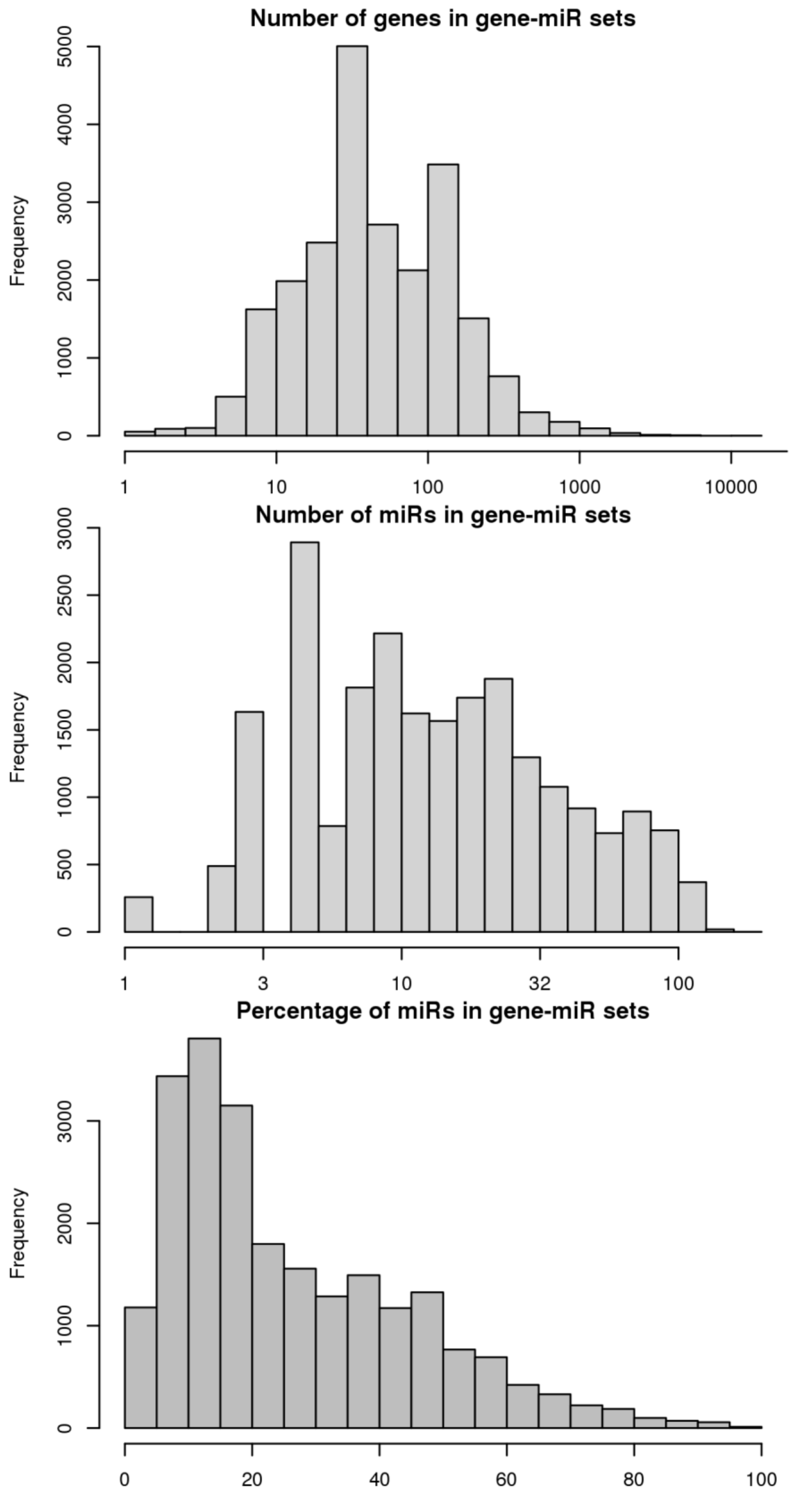

Supplementary Figure S3: Number of genes and miR sets in gene-miR sets. From up to down: (i) Number of genes in gene-miR sets, (ii) number of miRs in gene-miR sets, (iii) percentage of miRs in gene-miR sets.

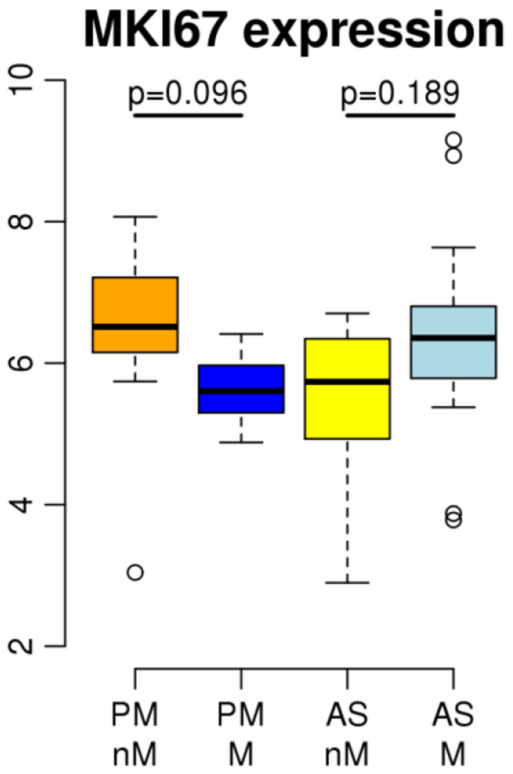

Supplementary Figure S4: Boxplot proliferation. Boxplot of MKI67 expression. Primary ovarian tumor cells (P) and metastatic peritoneal tumor cells (M) as well as ascitic single tumor cells (A) and spheroids (S) were summarized and analyzed for both tumor spread types non-miliary (nM) and miliary (M) separately. *P*-values were calculated with students' *T* test.

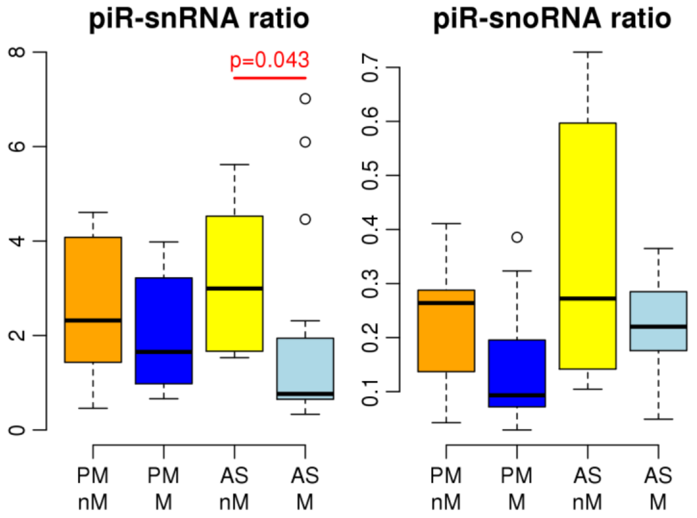

Supplementary Figure S5: Boxplot piRNAs. Boxplot of the amount of piRNAs; left: normalized to total small nuclear RNAs (piR-snRNA ratio) and right: normalized to total small nuclear RNAs (piR-snRNA ratio). Primary ovarian tumor cells (P) and metastatic peritoneal tumor cells (M) as well as ascitic single tumor cells (A) and spheroids (S) were summarized and analyzed for both tumor spread types non-miliary (nM) and miliary (M) separately. *P*-values were calculated with students' *T* test.

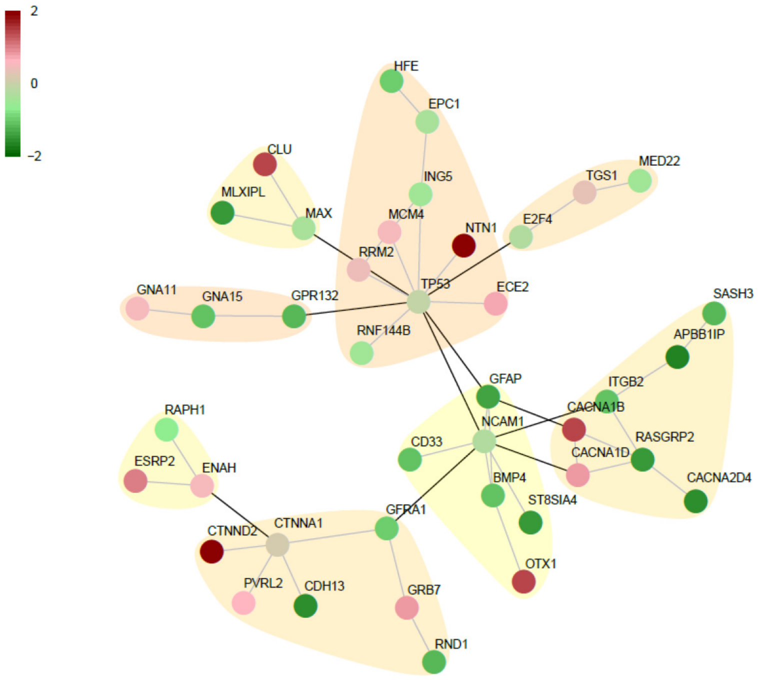

Supplementary Figure S6: High-scoring protein interaction network. The high-scoring protein-protein interaction network shows the interactions of reported and predicted targets of the four most deregulated miRNAs between miliary and non-miliary in solid tumors, miR-937–3p, novel-miR-1533, miR-1307–3p, and novel-miR-1294. Edges represent published evidence of interactions between two proteins (according to STRING 9.1 database). Red, upregulated and green, downregulated in miliary compared to non-miliary PM samples.

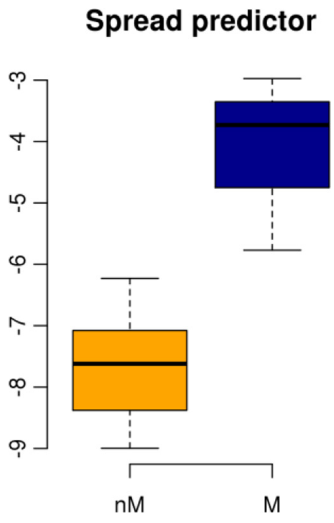

Supplementary Figure S7: Boxplot of the 13 sRNA spread predictor. Clear seperation of higher spread predictor values (calculated with the 13 sRNA signature from *sRNA-qPCR* data) of miliary (M) and lower spread predictor values of non-miliary (nM) samples of primary ovarian tumors (nM: n = 7, M: n = 3).

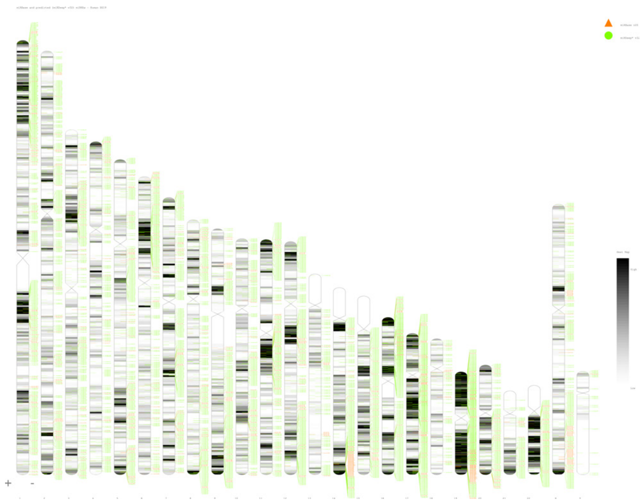

Supplementary Figure S8: miRNA loci on chromosomes (provided as extra picture). miRNA loci were visualized on human chromosomes with Idiographica: previously annotated miRNAs (miRBase v20) in orange and newly predicted miRNAs (miRDeep*) in green.
