# Supplementary material for: Small RNAs and the competing endogenous RNA network in high grade serous ovarian cancer tumor spread
Source: Oncotarget. 2016 May 9;7(26):39640–53. doi: 10.18632/oncotarget.9243 (PMC5129959; doi:10.18632/oncotarget.9243)
Supplement: Supplementary file 3 [file oncotarget-07-39640-s003.docx]

Supplementary Table S3: Known and novel predicted miRNAs and piRNAs analyzed in this study

| Comparison^1^ | Known | Novel | Total | Known | Sequence | Predicted | Sequence |
| --- | --- | --- | --- | --- | --- | --- | --- |
| PM: M vs nM | 10 [9]* | 12 [11]* | 21 | miR-200b-3p  miR-330–3p  miR-760  miR-937–3p  miR-1254  miR-1307  miR-3064–5p  miR-3188  miR-3940–3p  miR-191–5p (single assay) | UAAUACUGCCUGGUAAUGAUGA  GCAAAGCACACGGCCUGCAGAGA  CGGCUCUGGGUCUGUGGGGA  AUCCGCGCUCUGACUCUCUGCC  AGCCUGGAAGCUGGAGCCUGCAGU  ACUCGGCGUGGCGUCGGUCGUG  UCUGGCUGUUGUGGUGUGCAA  AGAGGCUUUGUGCGGAUACGGGG  CAGCCCGGAUCCCAGCCCACUU  CAACGGAAUCCCAAAAGCAGCUG | novel miR-804  novel miR-967  novel miR-1003  novel miR-1294  novel miR-1331  novel miR-1533  novel miR-1576  novel miR-1849  novel miR-1862  novel miR-2432  novel miR-2508  novel miR-2769 | CCGCGCCCCCGCCCCGGC  CGCCCCCCCGGUGUCCCC  GCGGCGGCGGCGGCGGUG  AAAGCAAAUGUUGGGUGAACGG  GAGGGAGCGGGCUCCGGCC  UGAAGCGCCUGUGCUCUGCCGAG  UGUGCAUUUCUCUCCCUUCUAG  CUAAGGGACCUUGGAGACAGGC  CUUCCCCACCCUCUCCUGCAG  ACCGGGUGCUGUGGCUUU  AAGUUUCUCUGAAAGUGUAGAG  GCGGCGGCGGCGGCGGCGA |
| M vs nM |  | 1 [1]* | 1 |  |  | novel miR-2916 (single assay) | CGCGCGCG[C/T]GTGTGGTGTG(T) |
| AS: M vs nM | – | 4 [3]* | 4 |  |  | novel miR-2353  novel miR-2364  novel miR-3475  novel miR-3784 | CCCCUCCUCCCCGCGC  CGCCCCCCGGCCCCGCG  UGGGGGCGGCGGCGGGGG  CGGGUCGGGGGGCGGG |
| AS vs PM | 4 [4]* | 5 [3]* | 9 | miR-143–3p  miR-214–3p (duplicated)  miR-2355–3p  miR-3177–3p | UGAGAUGAAGCACUGUAGCUC  ACAGCAGGCACAGACAGGCAGU  AUUGUCCUUGCUGUUUGGAGAU  UGCACGGCACUGGGGACACGU | novel miR-347  novel miR-1116  novel miR-1628  novel miR-1927  novel miR-3238 | CCGUGGACUGUGUGAGGCA  GCGGCUGGACGAGGCG  AGGCUGGGACUACAGGUG  CGCGCUCUCCCCCCUCCC  GGCUGGUCCGAUGGUAGU |
| normalizer | 4 [4]* |  | 4 | miR-92a-3p  miR-101–3p  miR-103a-3p (single assay)  miR-106b-5p (single assay) | UAUUGCACUUGUCCCGGCCUGU  UACAGUACUGUGAUAACUGAA  AGCAGCAUUGUACAGGGCUAUGA  UAAAGUGCUGACAGUGCAGAU |  |  |
| Total miRNAs | 18 [17]* | 22 [18]* | 40 [35]* |  |  |  |  |
| piRNAs | 5 [5] | 2 [2]* | 7 | piR-no_hsa_001101  piR-no_hsa_009294  piR-no_hsa_009295  piR-no_hsa_020450  piR-no_hsa_020548 | GUAAGUGAAGAUAAAGUGUGUCUGAGG  AUUGAUCAUCGACACUUCGAACGCACUUG  AUUGGUGGUUCAGUGGUAGAAUUCUCGC  GGAGAUGAAGAGGACAGUGACUGAGAGAC  GGUCAGUCGGUCCUGAGAGAUGGGCGAG | novel piR-n9_chr19_12814412  novel piR-n9_chr19_12817301 | GGAUGUAUUCGUACUGUCUGAUGGG  GGGAAGUGAUGACACCUGUGAC |

| normalizer |  | 1 [1]* | 1 |  |  | novel piR-n4_chr11_122017273 | UGAGGUAGUAGGUUGUAUAGUUAA |
| --- | --- | --- | --- | --- | --- | --- | --- |
| Total | 23 [22]* | 25 [21]* | 48 [43]* |  |  |  |  |

1PM: M vs nM, differentially expressed genes between miliary (M) and non-miliary (nM) in solid tumor samples (P and M).

M vs nM, differentially expressed genes between miliary (M) and non-miliary (nM) regardless of the analyzed tissues type.

AS: M vs nM, differentially expressed genes between miliary (M) and non-miliary (nM) in tumor cells of the ascites (A and S).

AS vs PM, differentially expressed genes between tumor cells from solid tumors(P and M) and tumor cells from ascites (A and S).

*[] number of expressed mi/piRNAs (as defined: Cq < 38 in > 75% samples), bold names highlight the non-expressed targets.
